# Effects of community health worker–led interventions on cardiometabolic outcomes in vulnerable populations: A systematic review and meta-analysis

**DOI:** 10.1371/journal.pgph.0007191

**Published:** 2026-08-26

**Authors:** Ismaila Ajayi Yusuf, Ihesiulo Alozie, Micah Nnabuko Okwah, Vikash Kumar, Zubeda Idris-Saeed, Hakeem Popoola, Emmanuel Babawale, Daniel Adepoju, Ayotunde Famokunwa, Omolaso Oluwabamise

**Affiliations:** 1 Department of Internal Medicine, Guthrie Robert Packer Hospital, Sayre, Pennsylvania, United States of America; 2 Department of Medicine, University of Calabar Teaching Hospital, Calabar, Nigeria; 3 Department of Epidemiology, Yale School of Public Health, New Haven, Connecticut, United States of America; 4 Department of Internal Medicine, Jinnah Sindh Medical University, Jinnah, Pakistan; 5 Department of Internal Medicine, Usmanu Danfodiyo University, Sokoto, Nigeria; 6 Department of Internal Medicine, Henry Ford Jackson Hospital, Jackson, Michigan, United States of America; 7 Department of Medicine, Obafemi Awolowo University, Ile-Ife, Nigeria; 8 Department of Health Informatics, Indiana University, Indiana, United States of America; 9 Department of Medicine, Obafemi Awolowo University, Ile-Ife, Nigeria; Universiti Malaya, MALAYSIA

## Abstract

Cardiometabolic diseases (CMDs) disproportionately affect vulnerable populations worldwide. Community health worker (CHW)–led interventions may reduce risk and improve preventive care, but their effectiveness remains unclear. This systematic review and meta-analysis evaluated their effectiveness on cardiometabolic outcomes in vulnerable groups. A comprehensive search of PubMed/MEDLINE, Scopus, Embase, ClinicalTrials.gov, and Cochrane Library was performed from January 1, 2000 to December 20, 2025, for randomized trials of CHW-led interventions on cardiometabolic outcomes. Primary outcomes were systolic and diastolic blood pressure; secondary outcomes were body mass index (BMI), glycosylated hemoglobin (HbA1c), and low-density lipoprotein cholesterol (LDL-C). Outcomes were pooled as mean differences using the generic inverse variance method; heterogeneity was assessed using I^2^. Thirty randomized trials (15,295 participants) were included. CHW-led interventions significantly improved systolic blood pressure (WMD = −3.72 mmHg; 95% CI: −5.94 to −1.51; I^2^ = 99.1%) and diastolic blood pressure (WMD = −2.22 mmHg; 95% CI: −3.50 to −0.95; I^2^ = 98.6%); 95% prediction intervals crossed the null for all five outcomes (e.g., SBP: −15.12 to 7.67 mmHg), indicating uncertainty at a new site. BMI and HbA1c improved modestly but were attenuated to non-significance by trim-and-fill analysis, suggesting publication bias; LDL-cholesterol effects were not significant. SBP reductions were significant in low- and middle-income countries (LMICs) (WMD = −5.09 mmHg; 95% CI: −7.57 to −2.60) but not high-income countries (HICs), with the largest effects in Africa. GRADE certainty was low for blood pressure, moderate for BMI, very low for HbA1c, and low for LDL-cholesterol. CHW-led interventions modestly improve cardiometabolic outcomes in vulnerable populations, with low-to-very-low certainty evidence for blood pressure and glycemic control and moderate certainty for BMI, supporting a potential role in cardiovascular risk-reduction strategies, particularly in LMICs. However, wide prediction intervals and non-significant meta-regression for intervention design features (contact frequency, duration, delivery format) warrant cautious, hypothesis-generating interpretation pending higher-quality trials.

**Systematic review Registration:**

**Registration Number:** PROSPERO 2025 CRD420251248842

## 1. Introduction

Cardiometabolic diseases (CMDs) refer to a group of related cardiovascular risk factors, such as hypertension, type 2 diabetes, obesity, dyslipidemia, and other metabolic disorders [1]. Collectively, these diseases adversely affect billions of people globally and continue to inflict significant morbidity and mortality. As of 2025, about 1.4 billion and 589 million people were estimated to be living with hypertension and diabetes, respectively, and these figures are projected to increase further by 2030 [2,3]. In addition, approximately 25% of the global adult population is estimated to have metabolic syndrome [4]. The implications of these are profound, as cardiovascular diseases, the complications of CMDs, account for almost 17.9 million deaths annually, about 1 in every 3 deaths [5]. Over 80% of these deaths occur in low and middle-income countries, due to poor healthcare access and technology for timely cardiovascular interventions. Besides causing substantial morbidity and mortality, cardiometabolic diseases also place a heavy burden on healthcare systems and the economy. Direct medical expenses attributed to CMDs in the US exceed $863 billion annually, and when indirect expenses, such as loss of job productivity and premature mortality, are considered, the total economic burden skyrockets [5].

The complications of CMDs are not evenly distributed globally. They disproportionately affect certain socially vulnerable groups with greater severity. These include populations in low- and middle-income countries, residents of isolated and rural communities, and ethnic and racial minority populations, as well as migrant and Indigenous populations and other groups affected by chronic structural socioeconomic marginalization [6–8]. People with lower socioeconomic status die of heart disease at rates significantly higher than their counterparts with higher socioeconomic status; studies indicate that these individuals are 50–80% more likely to develop heart disease. Even in developed nations, geographic location remains an important determinant of health outcomes. Residents of rural areas or regions distant from urban centers often experience substantial difficulties in obtaining regular health checkups, preventive care, and specialist services. As a result, diagnosis of CMDs is frequently delayed, which further exacerbates disease severity [6–9]. Over the years, healthcare systems have primarily delivered CMD prevention and treatment services through clinics and hospitals, although this approach has not yielded consistent success across populations [10].

To augment the traditional approach and curb the rising burden of CMDs, health programs designed within communities have gained traction in recent years. Rather than relying solely on hospitals and clinics, these programs bring care directly to people’s homes, workplaces, and communities. The interventions focus on change at the individual, group, and organizational levels and also support policy or environmental changes that encourage healthier behaviors [11,12]. They include programs delivered at non-clinical community venues such as community centers, places of worship, schools, or participants’ homes. The model is unique because, first, it relies primarily on non-physician community health workers, peer educators, or lay health advisors who share similar cultural and linguistic characteristics with the target population [10,13]. Second, the programs are patient-centered, grounded in the biopsychosocial model of behavioral change, and culturally sensitive, making participation more accessible and acceptable [14].

Despite the positive signals from community health worker–led interventions, most reviews have focused on hypertension or diabetes only. Although Jeet et al. (2017) previously examined CHW interventions for non-communicable disease prevention across diverse cardiometabolic outcomes, their review was restricted to LMICs and predates a substantial body of recent trial evidence. The present review extends Jeet et al. (2017) by incorporating newer evidence, including both LMIC and HIC settings to enable direct comparative subgroup analysis by national income classification, and synthesizing outcomes across the full spectrum of CMDs, including hypertension, obesity, dyslipidemia, and diabetes, in vulnerable populations globally [15–17]. Thus, this review aims to characterize CHW-led interventions in vulnerable populations, quantify the extent to which these programs have improved vital cardiometabolic endpoints, and determine which program characteristics are associated with better outcomes. The results will be vital for clinical practice, inform health policymakers, and aid the development of successful cardiometabolic disease prevention strategies.

## 2. Methods

**Fig 1:**
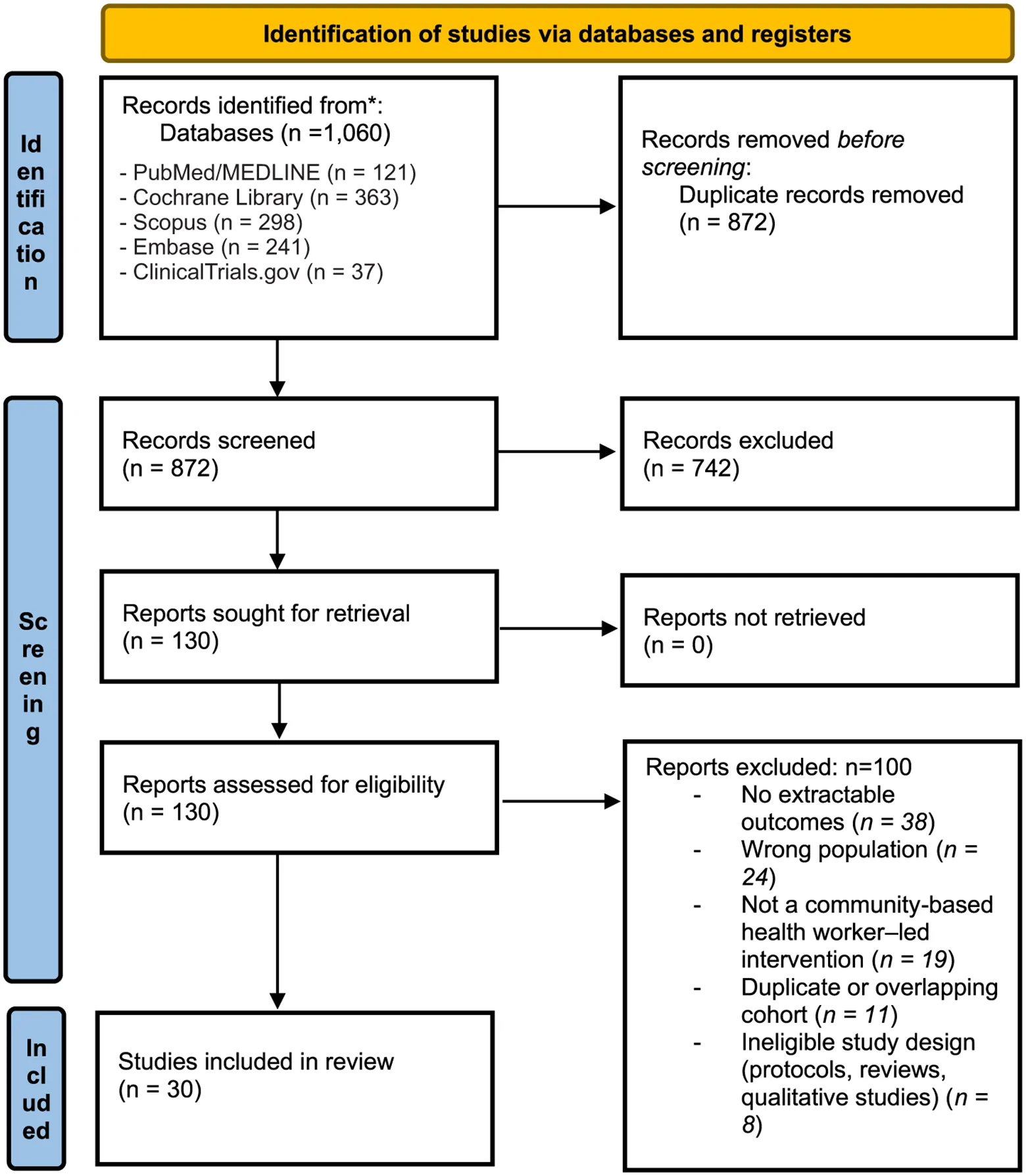
PRISMA Flowchart.

This meta-analysis was conducted according to the Cochrane Handbook and PRISMA (Preferred Reporting Items for Systematic Reviews and Meta-Analyses) guidelines [18,19]. Approval from the Institutional Review Board was not required as only publicly available data were used for the analysis (Fig 1).

### 2.1. Literature search and study selection

We extensively searched PubMed/MEDLINE, Scopus, Embase, ClinicalTrials.gov, and Cochrane Library from January 1, 2000 to December 20, 2025, for relevant studies. The search strategy included terms such as “community health worker” AND “cardiometabolic diseases” AND “community-based interventions.” A detailed search strategy for all included databases is provided in S1 and S2 Files. After retrieving the studies, duplicates were identified and removed using the EndNote reference management software, version 20.2.1 (Clarivate Analytics). The reference lists of retrieved trials, previous meta-analyses, and review articles were also examined to include additional relevant studies. Two independent authors (I.A.Y and I.A) conducted a thorough review of the remaining articles based on titles and abstracts, followed by a full-text review to select relevant studies. In case of any disagreement, a third reviewer (M.O) resolved the dispute.

### 2.2. Inclusion and exclusion criteria

Studies were included if they enrolled adults aged ≥18 years with cardiometabolic risk factors, including hypertension, diabetes mellitus, obesity, or dyslipidemia. Importantly, eligibility was determined by vulnerability status rather than national income classification; therefore, studies conducted in both low- and middle-income countries (LMICs) and high-income countries (HICs) were eligible when participants met predefined vulnerability criteria. Vulnerable or underserved populations were operationally defined, based on author-reported descriptions, as those meeting at least one of the following: (1) residence in a World Bank–classified LMIC; (2) self-identified racial or ethnic minority status; (3) low socioeconomic status (e.g., income below national poverty thresholds or equivalent); (4) residence in rural or geographically isolated communities with documented barriers to healthcare access; or (5) populations explicitly described as having limited healthcare access, low health literacy, or structural socioeconomic marginalization. To ensure comprehensive global coverage, database search strings were executed without any geographic, national income, or language restrictions, guaranteeing equal retrieval potential for studies from both high-income countries (HICs) and low- and middle-income countries (LMICs). In addition, eligible studies were required to be conducted in community-based or outpatient settings to ensure relevance to non-inpatient care contexts.

Studies were excluded if they involved participants younger than 18 years of age, were conducted exclusively in pregnant populations, or included populations without cardiometabolic risk factors. In addition, studies limited to inpatient or hospitalized settings only were excluded. Case reports, case series, editorials, commentaries, letters to the editor, and other non–original research articles were also excluded from the review.

### 2.3. Data extraction and quality assessment

**Fig 2:**
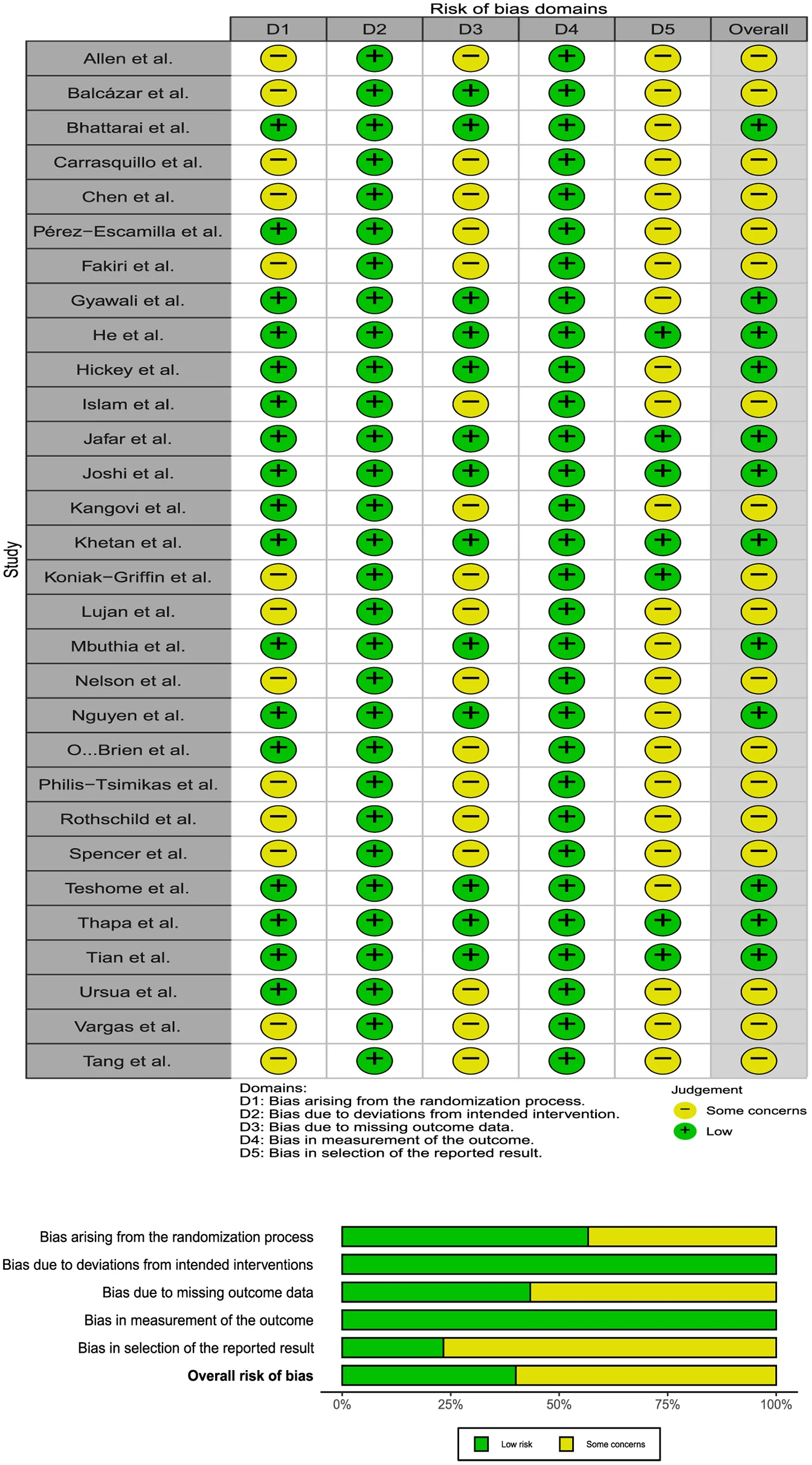
Risk of bias assessment (traffic light and summary plot).

Two independent reviewers (I.A.Y and I.A) independently extracted data from the included studies, including author name, study year, sample size, percentage of male participants, mean age, comorbidities, and relevant outcome variables. A third reviewer (M.O) checked the data extraction for accuracy, and any discrepancies were resolved through mutual discussion. The methodological quality of the included RCTs was assessed using the revised Cochrane Collaboration’s Risk of Bias tool (RoB-2) [19]. Risk of bias was assessed using the Cochrane RoB 2 tool across five domains: randomization process (sequence generation, allocation concealment, baseline imbalance, and allocation predictability), deviations from intended interventions (blinding, adherence, contamination/co-interventions, and analysis approach), missing outcome data (extent, reasons, balance, missingness mechanism, and handling methods), outcome measurement (blinding of assessors, objectivity, consistency/validity of measures, and influence of knowledge of intervention), and selection of the reported result (protocol availability, selective reporting of outcomes or time points, analytic flexibility, and outcome switching). Each study was graded as low risk, some concerns, or high risk based on domain-level judgments (Fig 2). Of the 30 included trials, 12 (40%) were judged overall low risk of bias and 18 (60%) some concerns; no trial was rated high risk. Risk of bias was most frequently elevated in domain 5 (selection of the reported result; 23/30 some concerns), while domains 2 (deviations from intended intervention) and 4 (outcome measurement) were rated low risk in all 30 trials.

The certainty of evidence for each primary outcome was assessed using the Grading of Recommendations Assessment, Development and Evaluation (GRADE) approach. Evidence from randomized controlled trials was initially rated as high certainty and downgraded based on risk of bias, inconsistency, indirectness, imprecision, and publication bias. GRADE ratings were independently assessed by two reviewers (I.A.Y and M.O), with discrepancies resolved by discussion. The overall certainty of evidence for each outcome was categorized as high, moderate, low, or very low. Findings are reported in the results section of this study.

### 2.4. Statistical analysis

Heterogeneity was assessed using the I² statistic [20] and first explored through prespecified subgroup analyses by geographic region (Africa, Asia, USA minorities) and national income classification (LMICs vs. HICs), followed by mixed-effects meta-regression to assess whether these variables explained between-study variance. Additional meta-regression models tested intervention delivery format (individual, group-based, mixed, remote/digital), intervention duration, and their interaction with national income classification as moderators of effect size for each outcome. Ninety-five percent prediction intervals were calculated for all pooled outcomes to estimate the range of effects expected in a new setting. For cluster-randomized trials, effect estimates and variances were used as reported by original trial investigators. To assess the robustness of the pooled estimates, leave-one-out sensitivity analyses were subsequently performed by systematically excluding one study at a time and re-estimating the pooled effect, allowing evaluation of whether any single study disproportionately influenced the overall result. Leave-one-out analyses are not intended as heterogeneity source-identification procedures. Publication bias was evaluated using funnel plot analysis and Egger’s regression test. Formal publication bias assessment using Egger’s regression test and trim-and-fill analysis was conducted only for outcomes and subgroups with ≥10 contributing studies, consistent with Cochrane Handbook guidance. For subgroups with fewer than 10 studies, results are reported as exploratory and formal bias testing was not performed.

## 3. Results

### 3.1. General characteristics, study design, and interventions

A total of 30 randomized trials, encompassing 15,295 participants, met the inclusion criteria and were included in the quantitative synthesis, as shown in Table 1 [21–50]. The included studies evaluated community health worker (CHW)–led interventions aimed at improving cardiometabolic risk factors across diverse populations, healthcare systems, and geographic regions. Sample sizes ranged from 34 participants to 2,312 participants [32,43]. All included studies were randomized controlled trials, including both individually randomized and cluster-randomized designs. CHW interventions commonly involved structured lifestyle counseling, health education, behavioral coaching, community- or home-based monitoring, and facilitated linkage to clinical care, delivered either independently or in coordination with primary care teams. Comparator groups typically received usual care or minimal educational interventions. Intervention duration ranged from 6 to 60 months (median 12 months) across included trials, with substantial additional variation in contact frequency and delivery format. Detailed characteristics of intervention delivery (format, contact frequency, duration) are provided in S4 File.

**Table 1:** General Characteristics of included randomized controlled trials.

Study (Author, Year)	Country / Region	Population	N	Intervention	Comparator	Follow-up
Allen et al., 2011	USA	Adults at high CVD risk	525	CHW + nurse-led CVD risk reduction	Usual care	12 mo
Balcázar et al., 2010	USA (Border region)	High-risk Hispanic adults	328	Promotora-led lifestyle program	Usual care	24 mo
Bhattarai et al., 2024	Nepal	Adults with hypertension	1,252	CHW-led BP management	Usual care	12 mo
Carrasquillo et al., 2017	USA	Latinos with T2DM	300	CHW cardiovascular risk intervention	Usual care	12 mo
Chen et al., 2025	China	Adults with T2DM	178	Community-based digital lifestyle intervention	Usual care	12 mo
Escamilla et al., 2015	USA	Latinos with T2DM	211	CHW-led structured diabetes program	Usual care	12 mo
Fakiri et al., 2008	Netherlands	Adults in deprived communities	275	Intensified preventive care	Usual care	24 mo
Gyawali et al., 2021	Nepal	Adults with T2DM	212	Female CHV-delivered intervention	Usual care	12 mo
He et al., 2017	Argentina	Low-income adults with hypertension	1,404	CHW-led multicomponent program	Usual care	18 mo
Hickey et al., 2025	Kenya & Uganda	Adults with hypertension	200	CHW-facilitated telehealth	Standard care	12 mo
Islam et al., 2023	USA	South Asian immigrants	291	CHW-integrated primary care	Usual care	12 mo
Jafar et al., 2009	Pakistan	Adults with hypertension	658	Community-based BP control	Usual care	24 mo
Joshi et al., 2019	India	Rural adults at CV risk	2,312	CHW cardiovascular risk reduction	Usual care	12 mo
Kangovi et al., 2017	USA	Adults with multimorbidity	302	CHW support intervention	Enhanced usual care	6 mo
Khetan et al., 2019	India	Adults with CV risk factors	527	CHW-based integrated care	Usual care	12 mo
Koniak-Griffin et al., 2015	USA	Latina women	194	CHW lifestyle behavior program	Usual care	12 mo
Lujan et al., 2007	USA	Mexican Americans with T2DM	176	Promotora diabetes education	Usual care	6 mo
Mbuthia et al., 2024	Kenya	Adults with hypertension	80	CHW home-based BP intervention	Usual care	6 mo
Nelson et al., 2017	USA	Low-income adults with T2DM	287	Low-intensity CHW support	Usual care	12 mo
Nguyen et al., 2024	Vietnam	Adults with uncontrolled HTN	649	Multicomponent CHW intervention	Usual care	12 mo
O’Brien et al., 2017	USA	Latinas with prediabetes	58	Promotora-led lifestyle program	Standard care	12 mo
Philis-Tsimikas et al., 2011	USA	High-risk Mexican Americans	207	Peer-led diabetes education	Standard care	12 mo
Rothschild et al., 2014	USA	Mexican Americans with T2DM	252	CHW diabetes management	Usual care	12 mo
Spencer et al., 2010	USA	African American & Latino adults	164	CHW diabetes intervention	Usual care	12 mo
Tang et al., 2014	USA	Adults with T2DM	116	Peer vs CHW DSME	Standard education	12 mo
Teshome et al., 2024	Ethiopia	Rural hypertensive adults	425	Health extension worker program	Usual care	12 mo
Thapa et al., 2023	Nepal	Adults with hypertension	1,352	Lifestyle intervention by CHWs	Usual care	60 mo
Tian et al., 2015	China & India	High CVD-risk adults	2,086	Simplified multifactorial care	Usual care	12 mo
Ursua et al., 2018	USA	Filipino Americans	240	CHW BP intervention	Usual care	6 mo
Vargas et al., 2024	USA	Adults at T2DM risk	34	Promotora metabolic intervention	Usual care	12 mo

### 3.2. Cardiometabolic conditions, geographic distribution, and population characteristics

The included studies were conducted across North America, South Asia, East and Southeast Asia, sub-Saharan Africa, Latin America, and the Middle East. Diabetes-focused and mixed cardiometabolic studies were frequently conducted in the United States, often among underserved or minority populations (e.g., *Allen et al., Kangovi et al., Ursua et al.*) [23,30,45]. In contrast, many hypertension-focused trials were conducted in low- and middle-income countries, particularly in South Asia and Africa (e.g., *Jafar et al., Khetan et al., Tian et al. and Thapa et al.*) where CHWs were embedded within primary healthcare systems [26,31,44,46].

Across studies, participants were predominantly adults with established cardiometabolic risk factors or diagnosed hypertension, diabetes, or prediabetes. Several trials specifically targeted socially or economically disadvantaged populations, including individuals with limited healthcare access or high baseline cardiometabolic risk, as observed in *Balcázar et al., Kangovi et al.,* and *Carrasquillo et al.* [33,34,45]. Baseline cardiometabolic control was generally suboptimal across studies. Primary outcomes in the included trials included hypertension (most common), diabetes and mixed cardiometabolic conditions [21–50]. The summary of synthesized outcomes is seen in Table 2.

**Table 2:** Effects of community health worker–led interventions on cardiometabolic outcomes.

Outcome	N Studies	Model	Pooled Effect (WMD)	95% CI	Heterogeneity (I^2^)	Sensitivity Analysis	Publication Bias (Egger)	Trim-and-Fill Adjusted Effect
**Systolic BP (mmHg)**	28	Random-effects	−3.72	−5.94 to −1.51	99.1%	Effect stable; no influential study	t = −0.49, p = 0.63	−1.55 (−4.07 to 0.97)
		Fixed-effect	−1.84	−1.92 to −1.77	99.1%	—	—	—
**SBP – Africa subgroup†**	3	Random-effects	−8.40	−14.42 to −2.37	84.9%	No influential study	Not performed†	—
**SBP – Asia subgroup†**	9	Random-effects	−4.00	−6.83 to −1.17	99.8%	No influential study	Not performed†	—
**SBP – USA subgroup**	15	Random-effects	−1.73	−4.99 to 1.53	98.2%	No influential study	t = −1.04, p = 0.30	—
**SBP – LMICs**	13	Random-effects	−5.09	−7.57 to −2.60	99.8%	—	z = −0.85, p = 0.39	—
**SBP – HICs**	15	Random-effects	−2.36	−5.61 to 0.89	99.03%	—	—	—
**Diastolic BP (mmHg)**	22	Random-effects	−2.22	−3.50 to −0.95	98.6%	Effect stable; no influential study	t = −0.27, p = 0.79	−0.63 (−2.13 to 0.87)
		Fixed-effect	−0.64	−0.72 to −0.56	98.6%	—	—	—
**Body Mass Index (kg/m**^2^)	17	Random-effects	−0.39	−0.66 to −0.12	98.6%	Effect stable; no influential study	t = −0.61, p = 0.554	−0.15 (−0.46 to 0.17)
		Fixed-effect	−0.19	−0.20 to −0.17	98.6%	—	—	—
**LDL-C (mg/dL)**	11	Random-effects	−0.83	−4.70 to 3.04	98.7%	Stable; no influential study	NS	−1.20 (−4.84 to 2.43)
		Fixed-effect	−0.28	−0.29 to −0.28	98.7%	—	—	—
**HbA1c (%)**	15	Random-effects	−0.31	−0.53 to −0.10	98.8%	Effect stable; no influential study	t = −0.94, p = 0.37	−0.03 (−0.30 to 0.24)
		Fixed-effect	−0.09	−0.11 to −0.08	98.8%	—	—	—

**†** Formal publication bias testing not performed for subgroups with <10 contributing studies per Cochrane Handbook guidance; findings are exploratory.

### 3.3. GRADE certainty of evidence

Using the GRADE framework, evidence from randomized trials was rated as low certainty for systolic and diastolic blood pressure, moderate certainty for body mass index, very low certainty for HbA1c, and low certainty for LDL-cholesterol. Inconsistency was downgraded for systolic blood pressure, diastolic blood pressure, and HbA1c given I^2^ values exceeding 98% that were not explained by tested moderators (delivery format, intervention duration, or their interaction with income classification). Inconsistency was not downgraded for BMI, where these same moderators explained 70–78% of between-study heterogeneity (p < 0.01), indicating that heterogeneity for this outcome was largely accounted for rather than unexplained. Imprecision was considered for outcomes with small effect sizes and confidence intervals spanning clinically marginal effects. Taken together, these ratings indicate that current evidence for CHW-led interventions warrants cautious interpretation pending higher-quality primary trials, particularly for blood pressure and glycemic outcomes (Table 3).

**Table 3:** GRADE certainty of evidence.

Outcome	No. of Studies (Participants)	Study Design	Risk of Bias	Inconsistency	Indirectness	Imprecision	Publication Bias	Overall Certainty
**Systolic blood pressure**	28 (14,867)	RCTs	Serious	Serious	Not serious	Not serious	Serious	**Low**
**Diastolic blood pressure**	22 (9,098)	RCTs	Serious	Serious	Not serious	Not serious	Serious	**Low**
**Body mass index**	17 (3,915)	RCTs	Serious	Not serious	Not serious	Serious	Serious	**Moderate**
**HbA1c**	15 (3,278)	RCTs	Serious	Serious	Not serious	Serious	Serious	**Very Low**
**LDL-cholesterol**	11 (2,556)	RCTs	Serious	Serious	Not serious	Serious	Not serious	**Low**

### 3.4. Effect of CHW-led interventions on systolic blood pressure

**Fig 3:**
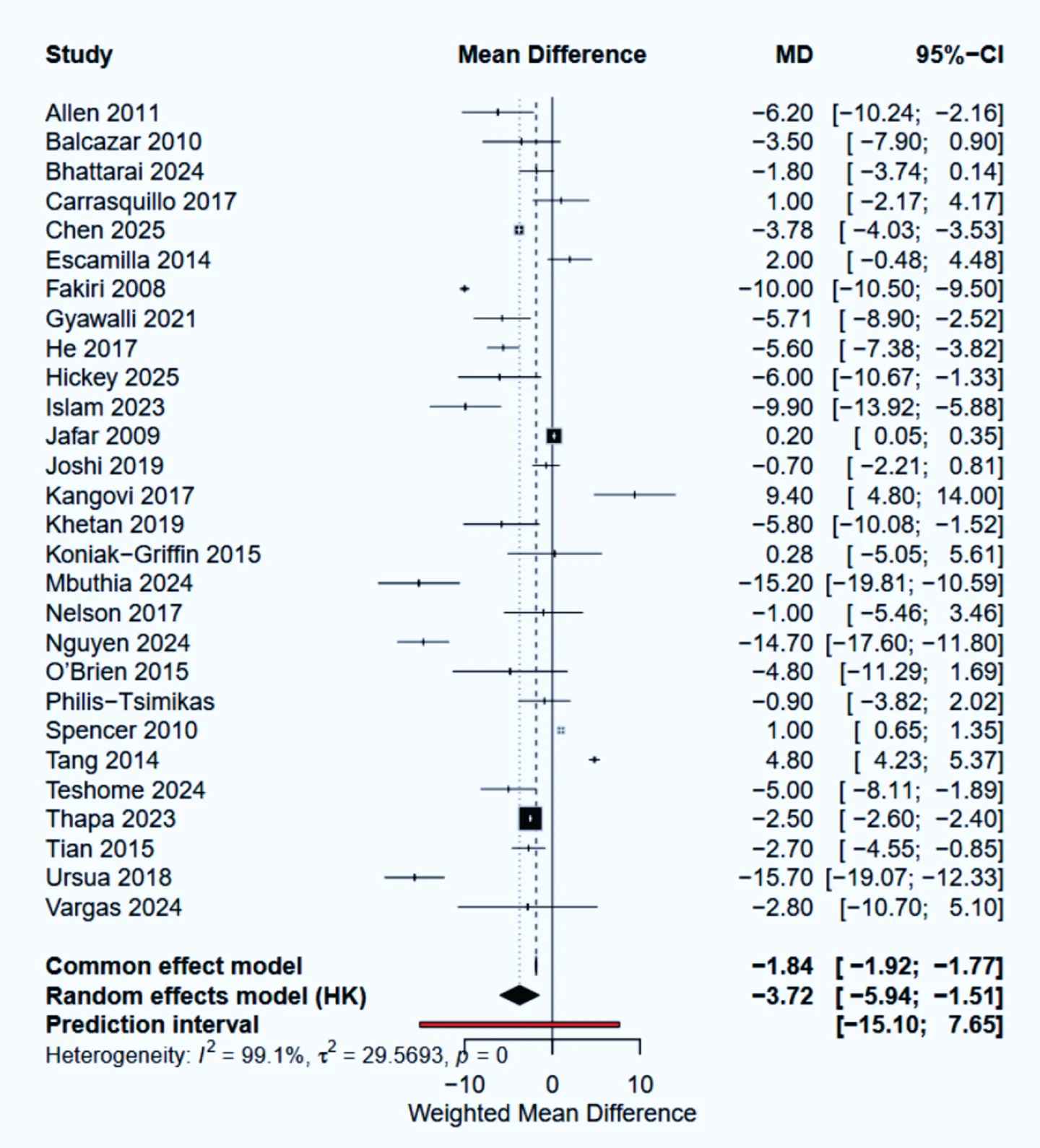
Forest plot for systolic blood pressure.

A forest plot of the weighted mean differences in systolic blood pressure is shown in Fig 3. Using a random-effects model, CHW-led interventions were associated with a statistically significant reduction in systolic blood pressure (WMD = −3.72 mmHg; 95% CI: −5.94 to −1.51; I^2^ = 99.1%). The 95% prediction interval was −15.12 to 7.67 mmHg. The fixed-effect model produced a smaller but more precise estimate (WMD = −1.84 mmHg; 95% CI: −1.92 to −1.77). Heterogeneity was substantial across all analyses; however, the direction of effect remained consistent, suggesting that variability reflects contextual differences in intervention delivery, population characteristics, and healthcare infrastructure rather than true inconsistency in treatment effect. To explore potential sources of heterogeneity, mixed-effects meta-regression was performed with geographic region, income classification (LMIC vs. HIC), and sample size as covariates. Sample size did not emerge as a significant moderator of the pooled SBP effect (p > 0.05), indicating that between-study heterogeneity was not explained by study size. Additional meta-regression testing delivery format (individual, group-based, mixed, remote/digital), intervention duration, and their interaction with national income classification found no significant moderators of SBP effect size (all omnibus p > 0.1). The GRADE certainty of evidence for systolic blood pressure was rated as low, downgraded from high due to serious risk of bias across most included trials, serious inconsistency (I^2^ = 99.1%, not explained by tested moderators including delivery format, duration, and income interaction), and potential publication bias as reflected in trim-and-fill attenuation.

Leave-one-out sensitivity analyses confirmed the robustness of the pooled estimate (S1 Fig). No single study, when excluded, materially altered the direction or statistical significance of the result, and I^2^ values remained close to 99% across all scenarios.

**Fig 4:**
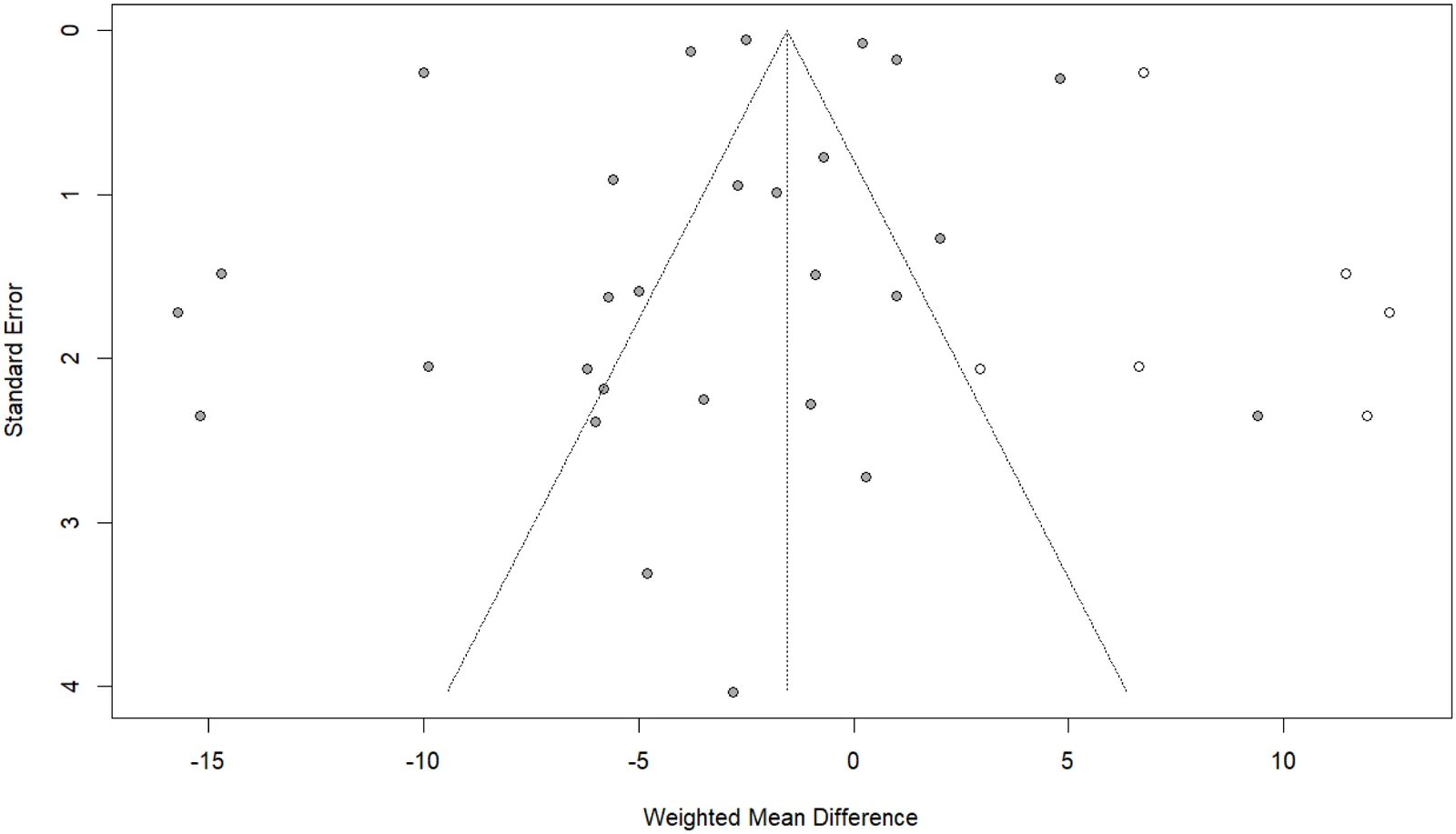
Funnel plot for assessment of publication bias in systolic blood pressure analysis.

Publication bias was assessed by visual inspection of the funnel plot (Fig 4) and Egger’s regression test. The Egger test did not indicate funnel plot asymmetry (t = −0.49, df = 26, p = 0.63). An exploratory trim-and-fill analysis imputing six hypothetical missing studies attenuated the pooled estimate to non-significance (WMD = −1.55 mmHg; 95% CI: −4.07 to 0.97), with heterogeneity remaining extremely high (I^2^ = 99.3%). Given the well-documented limitations of trim-and-fill in settings of substantial heterogeneity, these results are interpreted cautiously and do not override the primary Egger test finding (S2 Fig).

#### 3.4.1. Comparison of CHW-led interventions across geographic regions.

**Fig 5:**
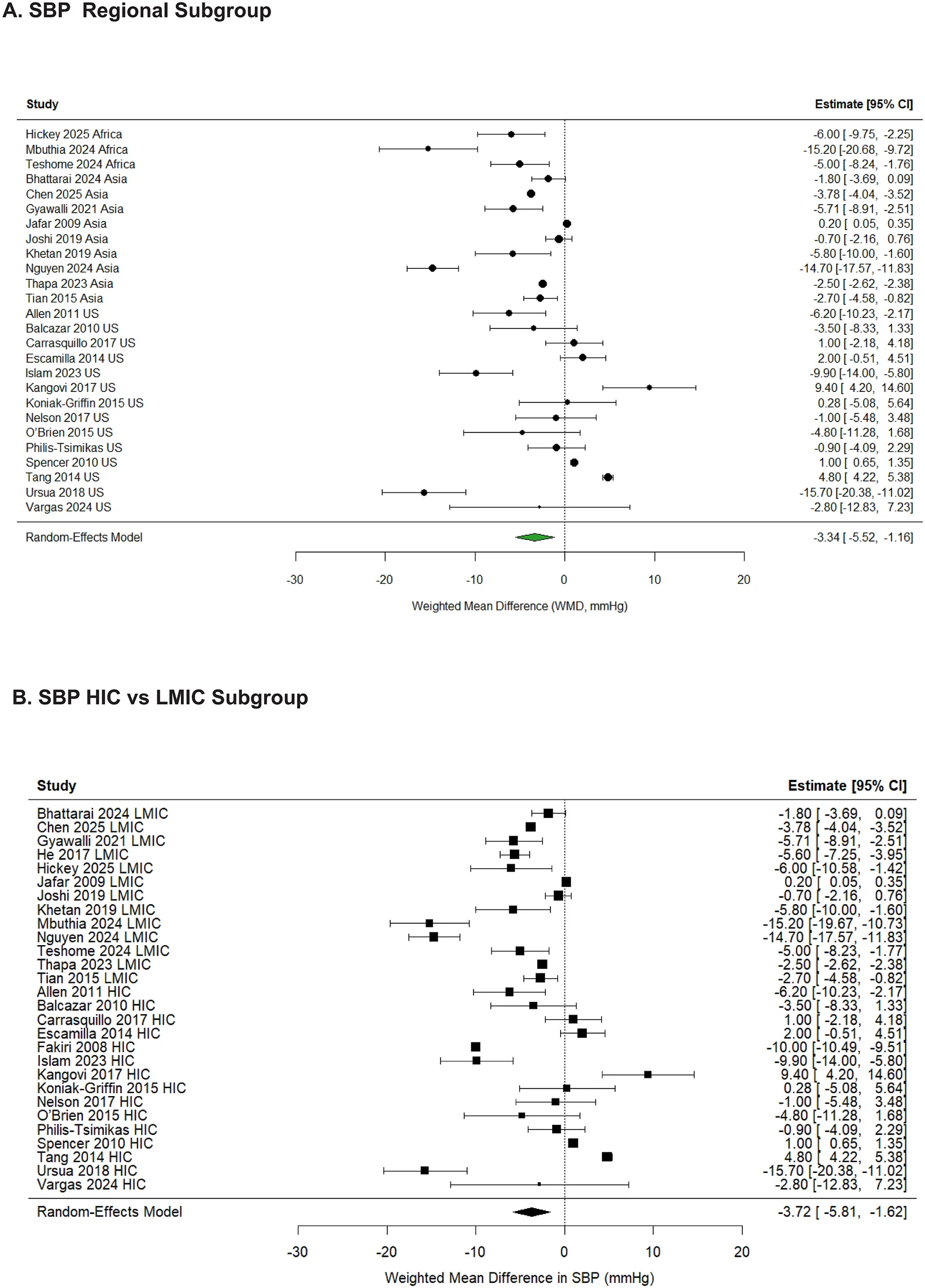
Panels A and B represent systolic blood pressure subgroup analyses by socioeconomic classification and geographic region.

Subgroup analyses by geographic region are shown in Fig 5. The largest SBP reduction was observed in African settings (WMD = −8.40 mmHg; 95% CI: −14.42 to −2.37; I^2^ = 84.9%; τ^2^ = 23.80), followed by Asian populations (WMD = −4.00 mmHg; 95% CI: −6.83 to −1.17; I^2^ = 99.8%; τ^2^ = 17.54), and USA minority populations (WMD = −1.73 mmHg; 95% CI: −4.99 to 1.53; I^2^ = 98.2%; τ^2^ = 33.46), the last of which did not reach statistical significance. Heterogeneity was high across all three subgroups.

Given that only three studies contributed to the Africa subgroup (Hickey 2025, Mbuthia 2024, Teshome 2024), formal publication bias testing was not performed for this subgroup; these findings should be interpreted as exploratory. For subgroups with ≥10 contributing studies, leave-one-out sensitivity analyses identified no single influential study, and Egger’s test showed no evidence of funnel plot asymmetry.

Mixed-effects meta-regression incorporating geographic region and sample size as covariates did not identify statistically significant effect modification by region (QM (df = 2) = 3.88, p = 0.144), and sample size was not a significant moderator of SBP effect within subgroups (p > 0.05). The consistency of effect direction and magnitude across regions nonetheless suggests that contextual factors meaningfully influence intervention effectiveness, even in the absence of formal statistical significance for between-subgroup differences (S2 and S3 Figs).

#### 3.4.2. Effectiveness of CHW-led interventions in low- and middle-income vs high-income countries.

Subgroup analysis by national income classification revealed a statistically significant SBP reduction in LMICs (WMD = −5.09 mmHg; 95% CI: −7.57 to −2.60; I^2^ = 99.79%; τ^2^ = 19.07), whereas the pooled effect in HICs did not reach statistical significance (WMD = −2.36 mmHg; 95% CI: −5.61 to 0.89; I^2^ = 99.03%; τ^2^ = 36.30). Heterogeneity was high in both subgroups, reflecting the diversity of intervention models, healthcare systems, and population characteristics within each income stratum rather than directional inconsistency. Mixed-effects meta-regression incorporating income classification and sample size as covariates did not demonstrate statistically significant effect modification by income group (QM = 1.77, df = 1, p = 0.18), and sample size was not a significant moderator of SBP effect in either stratum (p > 0.05). Income classification explained only a small proportion of between-study variance (R^2^ = 2.67%), suggesting that additional contextual factors beyond income status contribute to the observed heterogeneity. Nonetheless, the consistently larger and statistically significant pooled effect in LMICs — where baseline blood pressure is typically higher, CHWs are more deeply integrated into primary healthcare systems, and the marginal benefit of task-shifting is greater — is clinically meaningful and policy-relevant. Egger’s regression test did not indicate funnel plot asymmetry (z = −0.85, p = 0.39), providing no evidence of publication bias in this subgroup.

### 3.5. Effect of CHW-led interventions on diastolic blood pressure

**Fig 6:**
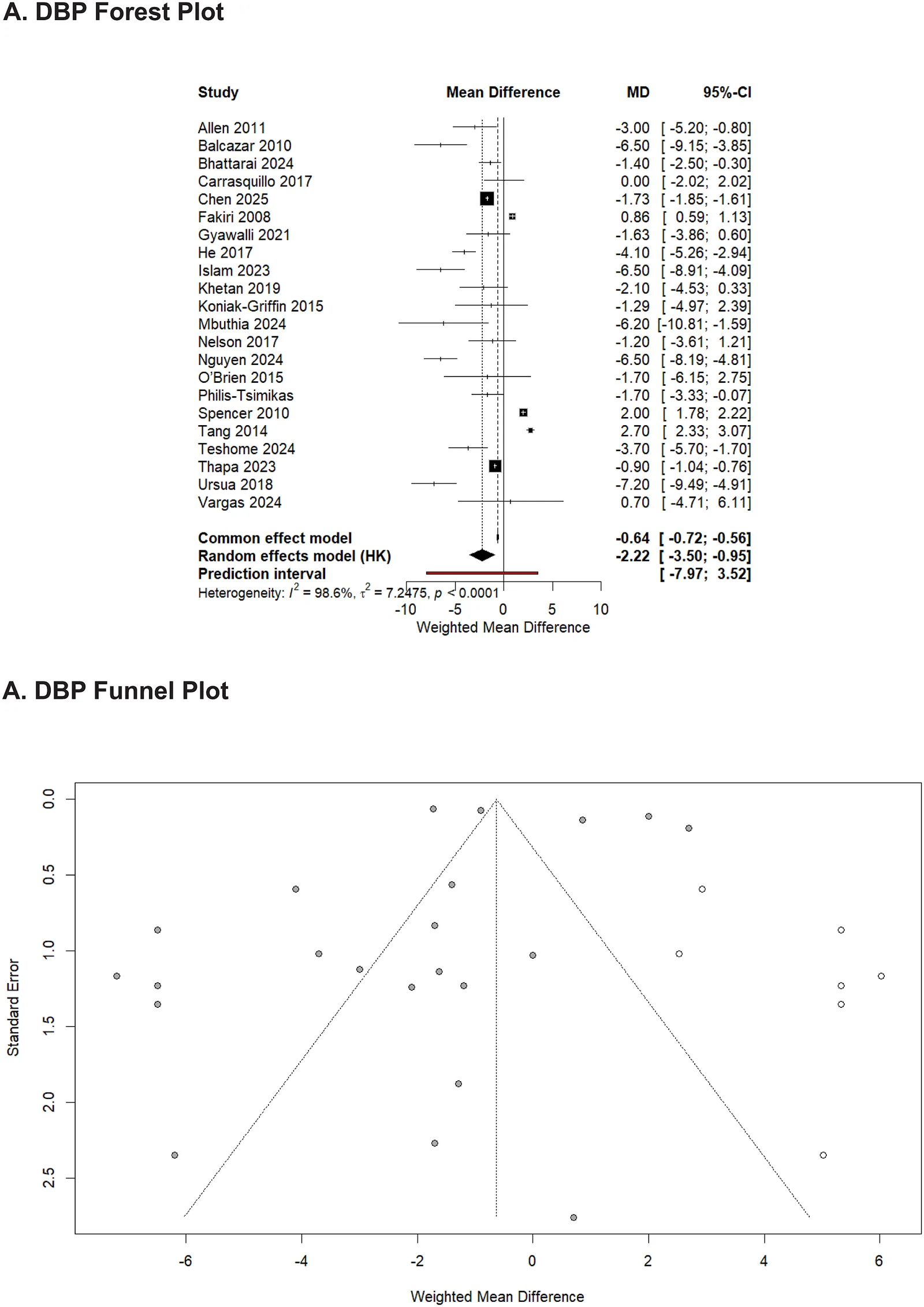
Forest plot for diastolic blood pressure.

A forest plot of the weighted mean differences in diastolic blood pressure is shown in Fig 6. Using a random-effects model, CHW-led interventions were associated with a statistically significant reduction in diastolic blood pressure (WMD = −2.22 mmHg; 95% CI: −3.50 to −0.95; I^2^ = 98.6%). The 95% prediction interval was −8.00 to 3.07 mmHg. The common-effect model produced a smaller, more precise estimate (WMD = −0.64 mmHg; 95% CI: −0.72 to −0.56). Leave-one-out sensitivity analyses confirmed the robustness of the pooled estimate; no single study, when excluded, materially altered the direction or statistical significance of the result, and I^2^ values exceeded 97% across all scenarios. Mixed-effects meta-regression did not identify sample size as a significant moderator of the DBP effect (p > 0.05), indicating that between-study heterogeneity was not attributable to study size. Additional meta-regression testing delivery format, intervention duration, and their interaction with income classification found no significant moderators of DBP effect size (all omnibus p > 0.1). Egger’s regression test did not indicate funnel plot asymmetry. However, exploratory trim-and-fill analyses suggested attenuation of the pooled effect, with heterogeneity remaining persistently high after adjustment, indicating potential sensitivity to unpublished or missing studies. Given the known limitations of trim-and-fill under conditions of substantial heterogeneity, these results are interpreted cautiously. The GRADE certainty of evidence for diastolic blood pressure was rated as low, downgraded from high due to serious risk of bias, serious inconsistency (I^2^ = 98.6%, not explained by tested moderators), and potential publication bias as reflected in trim-and-fill attenuation.

### 3.6. Effect of CHW-led interventions on body mass index

A forest plot of the weighted mean differences in BMI is shown in S4 Fig. Individual study effects were heterogeneous in magnitude; Chen et al. (2025), Fakiri et al. (2008), Tang et al. (2014), and O’Brien et al. (2015) demonstrated statistically significant BMI reductions favoring the intervention, whereas Nelson et al. (2017), Spencer et al. (2010), and Vargas et al. (2024) reported non-significant effects. Using a random-effects model, CHW-led interventions were associated with a statistically significant reduction in BMI (WMD = −0.39 kg/m^2^; 95% CI: −0.66 to −0.12; I^2^ = 98.6%). The 95% prediction interval was −1.28 to 0.51 kg/m^2^. The fixed-effect model produced a more precise estimate (WMD = −0.19 kg/m^2^; 95% CI: −0.20 to −0.17). Leave-one-out sensitivity analyses demonstrated a stable pooled BMI reduction with persistently high heterogeneity across all scenarios, and no single study exerted undue influence on the pooled estimate. Mixed-effects meta-regression did not identify sample size as a significant moderator of the BMI effect (p > 0.05), suggesting that between-study heterogeneity was not driven by study size. Additional meta-regression found that delivery format and intervention duration were significant moderators of BMI effect size, explaining a substantial proportion of between-study heterogeneity (R^2^ = 77.6% and 69.4% respectively, both p < 0.01). Egger’s test was non-significant; however, exploratory trim-and-fill analyses attenuated the pooled effect to non-significance, indicating potential sensitivity to missing or unpublished studies and warranting cautious interpretation of the BMI finding (S5 and S6 Figs). The GRADE certainty of evidence for BMI was rated as moderate, downgraded from high due to serious risk of bias, imprecision given the modest effect size and wide prediction interval, and potential publication bias as indicated by trim-and-fill attenuation.

### 3.7. Effect of CHW-led interventions on low-density lipoprotein cholesterol (LDL-C)

A forest plot of the weighted mean differences in LDL-cholesterol is shown in S7 Fig. The random-effects model showed a non-significant pooled effect (WMD = −0.83 mg/dL; 95% CI: −4.70 to 3.04; I^2^ = 98.7%). The 95% prediction interval was −12.71 to 11.06 mg/dL. The common-effect model produced a more precise but similarly non-significant estimate (WMD = −0.28 mg/dL; 95% CI: −0.29 to −0.28). Leave-one-out sensitivity analyses demonstrated a stable, non-significant pooled LDL-C effect with persistently high heterogeneity across all scenarios, confirming that no single study drove the null result. Mixed-effects meta-regression did not identify sample size as a significant moderator of the LDL-C effect (p > 0.05). Additional meta-regression testing delivery format, intervention duration, and their interaction with income classification found no significant moderators of LDL-C effect size. Egger’s test showed no evidence of publication bias. Exploratory trim-and-fill analysis imputing one hypothetical missing study produced no meaningful change to the adjusted pooled estimate (WMD = −1.20 mg/dL; 95% CI: −4.84 to 2.43), with heterogeneity remaining high (I^2^ = 98.6%), further confirming the absence of a reliable LDL-C effect (S8 and S9 Figs).

The null finding for LDL-C is clinically plausible, as lifestyle-focused community interventions delivered without concurrent pharmacologic lipid-lowering therapy are unlikely to produce meaningful reductions in LDL-C. The GRADE certainty of evidence for LDL-cholesterol was rated as **low**, downgraded from high due to serious risk of bias, serious inconsistency reflecting the wide confidence interval crossing the null, high between-study variance, and serious imprecision.

### 3.8. Effect of CHW-led interventions on glycated hemoglobin (HbA1c)

A forest plot of the weighted mean differences in HbA1c is shown in S10 Fig. Using a random-effects model, CHW-led interventions were associated with a statistically significant reduction in HbA1c (WMD = −0.31%; 95% CI: −0.53 to −0.10; I^2^ = 98.8%). The 95% prediction interval was −1.02 to 0.50%. The common-effect model produced a more precise estimate (WMD = −0.09%; 95% CI: −0.11 to −0.08). Leave-one-out sensitivity analyses demonstrated a stable pooled HbA1c reduction with persistently high heterogeneity across all scenarios, and no single study exerted undue influence on the pooled estimate. Mixed-effects meta-regression did not identify sample size as a significant moderator of the HbA1c effect (p > 0.05), indicating that between-study heterogeneity was not attributable to study size. Additional meta-regression testing delivery format, intervention duration, and their interaction with income classification found no significant moderators of HbA1c effect size. Egger’s test showed no evidence of publication bias. Exploratory trim-and-fill analyses attenuated the pooled effect without reducing heterogeneity, suggesting potential sensitivity to missing or unpublished studies and warranting cautious interpretation of the magnitude of the glycemic benefit (S11 and S12 Figs). The GRADE certainty of evidence for HbA1c was rated as very low, downgraded from high due to serious risk of bias, serious inconsistency (I^2^ = 98.8%, not explained by tested moderators), serious imprecision given the modest effect size and wide prediction interval, and potential publication bias as reflected in trim-and-fill attenuation.

## 4. Discussion, limitations, and conclusion

### 4.1. Discussion

Our systematic review and meta-analysis of 30 randomized controlled trials involving approximately 15,295 participants focused on cardiometabolic outcomes of CHW-led interventions. We found that these interventions were associated with improved outcomes across several cardiometabolic parameters. Notably, there was a more consistent and statistically significant reduction in both systolic and diastolic blood pressure across diverse geographic regions and income settings. At the same time, there was also a modest improvement in glycemic control and body mass index, although LDL-cholesterol was not statistically significant. Our results support previous findings in the literature that CHW-led interventions may serve as scalable adjuncts to usual care in improving cardiovascular risk factors [51].

The observed overall decrease in systolic blood pressure of approximately 4 mmHg is consistent with findings from a systematic review and meta-analysis of team-based care interventions involving community health workers, which revealed an average of 4.4 mmHg reduction in systolic blood pressure, consistent with modest but meaningful population-level improvements [52]. Anand et al.’s findings showed that CHWs’ task-sharing interventions reduced systolic blood pressure by 3.7 mmHg, whereas Jeet et al. demonstrated a reduction of 4.80 mmHg in LMICs [14,53]. A large meta-analysis of 344,716 participants evaluating the degree of blood pressure reduction that can reduce cardiovascular disease highlighted that a slight average reduction in systolic blood pressure of 5 mm Hg reduces the risk of cardiovascular disease events by 10% [54]. Similarly, Ettehad et al.’s systematic review and meta-analysis found that a 10 mm Hg reduction in systolic blood pressure reduced the risk of major cardiovascular disease events by 20%, underscoring the public health relevance of the effect size observed in this review [55].

Beyond pooled effects, an important contribution of this review is the demonstration of meaningful heterogeneity in systolic blood pressure reduction across geographic regions and income classifications. Subgroup analyses revealed the largest reductions in systolic blood pressure in African settings, followed by Asian populations, with smaller and non-significant effects observed among minority populations in the United States. Similarly, when stratified by national income status, low- and middle-income countries (LMICs) demonstrated a statistically significant and clinically meaningful reduction in systolic blood pressure, whereas high-income countries (HICs) did not. Although formal meta-regression did not identify statistically significant effect modification, the consistency of effect direction and magnitude across these subgroup analyses suggests that contextual factors—rather than random variability alone—may meaningfully influence intervention effectiveness [31–50].

Because formal meta-regression did not identify region or income classification as statistically significant effect modifiers, the following explanations are speculative and hypothesis-generating rather than confirmed mechanisms. Several plausible, though unconfirmed, factors could contribute to the numerically stronger blood pressure reductions observed in LMICs and certain geographic regions. First, baseline blood pressure levels are often higher and rates of untreated or undertreated hypertension are greater in resource-limited settings, which could allow for greater absolute reductions with even modest interventions [1–4]. Second, community health workers in LMICs are frequently embedded within primary healthcare systems and operate with defined scopes of practice, including blood pressure monitoring, medication adherence support, and referral pathways, which could enhance intervention fidelity. Third, task-shifting approaches may fill gaps in healthcare access where physician density is low, potentially yielding a greater marginal benefit of CHW-led care. In contrast, in HICs where access to healthcare may already be relatively higher, CHW interventions may function primarily as supportive or complementary services, which could account for the smaller incremental effects observed [1–4,56]. These proposed explanations warrant direct testing in future studies designed specifically to evaluate effect modification by setting.

The policy implications of these findings are substantial. Hypertension remains the leading modifiable risk factor for cardiovascular morbidity and mortality globally, with the greatest burden borne by LMICs. Our results suggest that scaling community health worker–led interventions in these settings could yield meaningful population-level blood pressure reductions with relatively low resource investment. Given that even small shifts in mean systolic blood pressure can translate into large reductions in cardiovascular events at the population level, the observed reductions in this meta-analysis are likely to be clinically and economically impactful. These findings support the integration of CHWs into national hypertension control strategies, particularly within primary care and community-based prevention frameworks aligned with World Health Organization HEARTS and similar initiatives [1–4].

In addition to blood pressure outcomes, this review identified modest and statistically significant improvements in body mass index and glycemic control. Although these effect sizes were smaller and attenuated after trim-and-fill analyses, they nonetheless suggest that CHW-led interventions can influence multiple cardiometabolic risk factors simultaneously. Such multidimensional benefits are particularly relevant in vulnerable populations where cardiometabolic conditions often cluster, and healthcare access is fragmented. In contrast, no significant effect was observed for LDL-cholesterol, which may reflect the limited role of lifestyle-focused community interventions in modifying lipid profiles without concurrent pharmacologic therapy. This finding underscores the importance of integrating CHW-led programs with formal healthcare systems capable of initiating and intensifying lipid-lowering treatment when indicated [57,58].

High statistical heterogeneity was observed across all outcomes, a common feature of complex, multi-component behavioral and health system interventions implemented across diverse contexts. Importantly, heterogeneity persisted despite sensitivity analyses and subgroup stratification, while the direction of effect remained largely consistent. This pattern suggests that heterogeneity likely reflects inherent contextual variability in intervention delivery, population characteristics, baseline risk, and healthcare infrastructure rather than inconsistency or lack of efficacy. From a policy perspective, this pattern is consistent with the intervention’s effects being context-dependent and potentially optimizable through local adaptation; nonetheless, the magnitude of unexplained heterogeneity (I^2^ 96–99%) remains a substantive limitation that constrains confidence in the pooled effect estimates and should be weighed accordingly when interpreting these findings.

### 4.2. Limitations and future directions

While the inclusion of 30 RCTs across multiple regions provides broad geographic representation, the substantial between-study heterogeneity (I^2^ 96–99% across outcomes) limits the generalizability of pooled effect estimates to any single setting; this is further underscored by prediction intervals that crossed the null for all five outcomes. The presence of heterogeneity due to the diversity of populations and interventions can impact the precision of the pooled estimates [20]. Differences in intervention intensity, CHW training, scope of practice, supervision models, healthcare integration, and baseline cardiometabolic risk likely contributed to variability in effect sizes [10–13,16,58]. While subgroup analyses demonstrated a consistent direction of effect across RCTs, the presence of high between-study heterogeneity limits generalizability across different settings.

Follow-up durations in the studies varied, ranging from 6 to 24 months, with a few extending beyond this. Cardiometabolic diseases are chronic in nature, requiring long-term care [1–5]. As a result, the follow-up durations may not capture the long-term outcomes such as improvements in blood pressure, glycemic control, and BMI. While decreases in blood pressure are useful indicators of cardiovascular risk reductions at the population level [54,55], few included studies assessed more specific cardiovascular endpoints such as hospitalization or mortality. Future trials should incorporate longer follow-up to determine whether observed improvements are sustained; existing evidence within this review (e.g., attenuated BMI effects in longer-duration trials, and the COBIN trial’s loss of benefit at 5-year follow-up) suggests treatment effects may not persist after intervention cessation, and meta-regression confirmed intervention duration was not associated with larger effects for SBP, DBP, HbA1c, or LDL. Despite the use of randomized trials in this study, most studies were unblinded, which could introduce bias. The biochemical nature of most outcomes compensates for the lack of blinding, as those outcomes are unlikely to be influenced by this limitation [19,20].

Future research should prioritize longer follow-up, employ standardized methods to report interventions, and ensure the roles of CHWs within the health system are better specified [10,13,58]. To identify factors that enhance effectiveness in LMICs, especially those where task-sharing has been established, implementation science frameworks and pragmatic trials are needed [16,53].

### 4.3. Conclusion

This systematic review and meta-analysis of 30 RCTs demonstrates that community health worker-led interventions resulted in moderate improvements in key cardiovascular risk factors among vulnerable populations. Significant reductions were noted in systolic and diastolic blood pressure, glycemic control, and BMI, with no significant improvement in LDL-cholesterol. Overall, this study demonstrated a positive impact of CHW-led programs in improving cardiovascular outcomes when combined with standard care.

Among LMICs, the observed reduction in blood pressure was both statistically and clinically significant, which is crucial because LMICs have the highest burden of hypertension. Subgroup analyses demonstrated significantly greater SBP reduction in LMICs than HICs, with the largest effects in African settings, suggesting that national income classification and regional health system context — rather than specific intervention design characteristics, which were not significant moderators in meta-regression — account for much of the observed variability in effectiveness. Given predominantly low to very low certainty evidence across outcomes, these findings should be interpreted as hypothesis-generating rather than practice-changing pending higher-quality primary trials. Given the favorable effects observed across multiple cardiometabolic outcomes, CHW-led interventions may serve as an important component of community-based cardiovascular risk reduction programs.

### 4.4. Ethics approval and consent to participate

Ethical approval and informed consent were not required for this study because it is a systematic review of previously published studies.

## Supporting information

S1 File PROSPERO registration document. (PDF)

S2 File Search strategy. (DOCX)

S3 File PRISMA checklist. This checklist is licensed under the Creative Commons Attribution License (CC BY 4.0), based on: Page MJ, McKenzie JE, Bossuyt PM, et al. The PRISMA 2020 statement: an updated guideline for reporting systematic reviews. BMJ. 2021;372:n71.
(DOCX)

S4 File Intervention characteristics. (PDF)

S5 File Extracted dataset. (XLSX)

S1 Fig SBP sensitivity analyses. (PNG)

S2 Fig Funnel plot of SBP by geographical regions. (PNG)

S3 Fig Funnel plot of SBP by socioeconomic regions. (PNG)

S4 Fig BMI forest plot. (PNG)

S5 Fig BMI funnel plot. (PNG)

S6 Fig BMI sensitivity analyses. (PNG)

S7 Fig LDL forest plot. (PNG)

S8 Fig LDL funnel plot. (PNG)

S9 Fig LDL sensitivity analyses. (PNG)

S10 Fig HbA1C forest plot. (PNG)

S11 Fig HbA1C funnel plot. (PNG)

S12 Fig HbA1C sensitivity analyses. (PNG)
