# Supplementary material for: Effects of community health worker–led interventions on cardiometabolic outcomes in vulnerable populations: A systematic review and meta-analysis
Source: PLOS Glob Public Health. 2026 Aug 26;6(8):e0007191. doi: 10.1371/journal.pgph.0007191 (PMC13514067; doi:10.1371/journal.pgph.0007191)
Supplement: Supplementary file 1 [file pgph.0007191.s001.pdf]

NIHR | National Institute for Health and Care Research

PROSPERO
International prospective register of systematic reviews

Can Community-Based Health Interventions Improve Cardiometabolic Outcomes in Vulnerable Populations? A Global Systematic Review and Meta-Analysis of Randomized Controlled Trials

Ismaila Yusuf, Hakeem Popoola, Micah Okwah

Citation
Ismaila Yusuf, Hakeem Popoola, Micah Okwah. Can Community-Based Health Interventions Improve Cardiometabolic Outcomes in Vulnerable Populations? A Global Systematic Review and Meta-Analysis of Randomized Controlled Trials. PROSPERO 2025 CRD420251248842. Available from https://www.crd.york.ac.uk/PROSPERO/view/CRD420251248842.

REVIEW TITLE AND BASIC DETAILS

Review title

Can Community-Based Health Interventions Improve Cardiometabolic Outcomes in Vulnerable Populations? A Global Systematic Review and Meta-Analysis of Randomized Controlled Trials

Condition or domain being studied

Essential Hypertension; Type 2 Diabetes Mellitus; Cardiometabolic comorbidities ; Primary Prevention Of Cardiovascular Disease; Healthcare access; Community-centred approach

Rationale for the review

Cardiometabolic diseases, including hypertension and diabetes, are the leading causes of morbidity and mortality worldwide, with a disproportionate burden borne by vulnerable populations in low- and middle-income countries and among racial and ethnic minorities in high-income settings. Despite the availability of effective pharmacologic and lifestyle-based treatments, persistent gaps in access to care, health literacy, medication adherence, and continuity of follow-up continue to limit optimal cardiometabolic control. Community-based health worker interventions have emerged as a scalable strategy to address these barriers by delivering culturally tailored, locally accessible, and patient-centered care. However, existing evidence remains fragmented across regions and outcomes, with uncertainty regarding the consistency and magnitude of their effects on key cardiometabolic endpoints. A comprehensive synthesis of randomized controlled

trials is therefore needed to quantify the impact of community-based health worker interventions on blood pressure, glycemic control, adiposity, and lipid profiles in vulnerable populations and to inform health system and policy decisions.

Review objectives

Primary Objective

- To determine the effect of community-based health worker interventions on systolic and diastolic blood pressure among vulnerable adult populations.

Secondary Objectives

- To evaluate the impact of these interventions on glycemic control (HbA1c), body mass index (BMI), and low-density lipoprotein cholesterol (LDL).

- To compare the effectiveness of community-based health worker interventions across geographic regions (Africa, Asia, Latin America, and the United States).

- To examine differences in intervention effects between low- and middle-income countries and high-income countries, including U.S. racial and ethnic minority populations.

- To assess the robustness of findings through predefined subgroup and sensitivity analyses.

Keywords

Cardiometabolic; Community health interventions; Vulnerable populations; Diabetes; Hypertension; Obesity; Hyperlipidemia

Country

United States of America; Nigeria; Pakistan

ELIGIBILITY CRITERIA

Population

Included

- Adults ≥18 years

- Vulnerable populations (low-income, minority, rural, underserved, LMICs)

- With cardiometabolic risk (hypertension, diabetes, obesity, dyslipidemia)

Excluded

- Studies population < 18 years of age,

- Pregnancy-only studies or studies in population without cardiometabolic risks

- Inpatient-only samples

Intervention(s) or exposure(s)

Included

- Community-based health worker-led interventions (CHWs, promotores, lay workers)

- Lifestyle, BP/diabetes management, adherence, or behavioral support

Excluded
- Hospital-only programs without community interventions

- Pharmacologic-only trials

Comparator(s) or control(s)

Included
Usual care / standard care / minimal intervention

Excluded
Another structured community-based intervention or equivalent active program

Study design

Only randomized study types will be included.

Context

Eligible studies must be conducted in community-based settings, including homes, neighborhoods, primary care–linked community programs, faith-based centers, or other non-hospital community venues. Interventions must be delivered primarily by community-based health workers (e.g., CHWs, promotores, lay or peer health workers) with direct interaction with participants.

Studies conducted exclusively in inpatient or hospital-only settings without community follow-up are excluded. No restrictions are placed on geographic region, country income level, or healthcare system.

Only randomized controlled trials (individual or cluster) with sufficient quantitative data for extraction of at least one cardiometabolic outcome are eligible. Studies must involve adult participants (≥18 years) from vulnerable or underserved populations and report outcomes related to blood pressure, glycemic control, adiposity, or lipid measures.

TIMELINE OF THE REVIEW

Date of first submission to PROSPERO

08 December 2025

Review timeline

Start date: 14 December 2025. End date: 17 January 2026.

Date of registration in PROSPERO

08 December 2025

AVAILABILITY OF FULL PROTOCOL

Availability of full protocol

A full protocol has been written but is not available because:

The full protocol has been written and undergoing final editing by the authors. We will make it available when it is completed.

SEARCHING AND SCREENING

Search for unpublished studies

Both published and unpublished studies will be sought.

Main bibliographic databases that will be searched

The main databases to be searched are *CENTRAL - Cochrane Central Register of Controlled Trials*, *CINAHL - Cumulative Index to Nursing and Allied Health Literature*, *Embase - Embase via Ovid*, *Embase.com*, *MEDLINE*, *PubMed* and *Scopus*.

Search language restrictions

The review will only include studies published in English.

Search date restrictions

Databases will be searched for articles published from 1 January 2005 and before by 12 December 2025.

Other methods of identifying studies

Other studies will be identified by: *contacting authors or experts*, *looking through all the articles that cite the papers included in the review ("snowballing" or forward citation searching)*, *reference list checking (backward citation searching)*, *searching conference proceedings*, *searching dissertation and thesis databases* and *searching trial or study registers*.

Link to search strategy

A full search strategy has been uploaded to PROSPERO. The PDF may be accessed through this link https://www.crd.york.ac.uk/PROSPEROFILES/34a085db6e5485072c4230e4d6a6c300.pdf.

Selection process

Studies will be screened independently by at least two people (or person/machine combination) with a process to resolve differences.

Other relevant information about searching and screening

None

DATA COLLECTION PROCESS

Data extraction from published articles and reports

Data will be extracted independently by at least two people (or person/machine combination) with a process to resolve differences.

Authors will be asked to provide any required data not available in published reports.

Study datasets/IPD will be obtained from study investigators or via a data repository

Study risk of bias or quality assessment

Risk of bias will be assessed using: *Cochrane RoB-2*

Data will be assessed independently by at least two people (or person/machine combination) with a process to resolve differences.

Additional information will be sought from study investigators if required information is unclear or unavailable in the study publications/reports.

Reporting bias assessment

Risk of bias due to missing results will be assessed

Certainty assessment

The certainty of evidence for each primary and secondary outcome will be assessed using the GRADE (Grading of Recommendations Assessment, Development and Evaluation) approach. Evidence will be rated as high, moderate, low, or very low based on the following five domains:

Risk of bias,

Inconsistency (heterogeneity),

Indirectness,

Imprecision, and

Publication bias.

OUTCOMES TO BE ANALYSED

Main outcomes

- Change in systolic blood pressure (SBP)

- Change in Diastolic Blood Pressure (DBP)

Additional outcomes

- Change in HbA1C (%)

- Change in Body Mass Index (BMI), kg/m2

- Change in LDL Cholesterol (LDL-c)

PLANNED DATA SYNTHESIS

Strategy for data synthesis

- STRATEGY FOR DATA SYNTHESIS

Data will be combined using the random-effects meta-analysis

Mean difference (MD) or standardized mean difference (SMD) with 95% CI will be obtained

Heterogeneity will be assessed using I^2 statistic

Forest plots will be generated for each outcome

- SUBGROUP ANALYSES WILL BE DONE AS BELOW:

Geographic region (LMIC vs HIC)

Study quality (low vs high risk of bias)

US Minorities vs NON-US Majority populations

Pooled response across different minority populations

- SENSITIVITY ANALYSIS

Exclusion of high-risk-of-bias studies

Fixed-effect vs random-effects comparison

CURRENT REVIEW STAGE

Stage of the review at this submission

Review stage	Started	Completed
Pilot work		
Formal searching/study identification		
Screening search results against inclusion criteria		
Data extraction or receipt of IPD		
Risk of bias/quality assessment		
Data synthesis		

Review status

The review is currently planned or ongoing.

Publication of review results

Results of the review will be published.

REVIEW AFFILIATION, FUNDING AND PEER REVIEW

Review team members

Dr Ismaila Yusuf (review guarantor and contact) ORCID: 0009-0002-2319-3899. Robert Packer Hospital, Guthrie. United States of America.

No conflict of interest declared.

Dr Hakeem Popoola. Henry Ford Jackson Hospital. United States of America.

No conflict of interest declared.

Dr Micah Okwah. Yale University. United States of America.

No conflict of interest declared.

Named contact

Dr Ismaila Yusuf (ismaila.yusuf@guthrie.org). ORCID: 0009-0002-2319-3899. Robert Packer Hospital, Guthrie. United States of America.

Review affiliation

Guthrie Robert Packer Hospital, USA

Yale University, USA

Henry Ford Jackson Hospital, USA

Funding source

Review has no funding and no agreed support from an academic institution and is done in authors' own time.

Peer review

There has been no peer review of this planned review.

ADDITIONAL INFORMATION

Review conflict of interest

Declared individual interests are recorded under team member details.. No additional interests are recorded for this review.

Medical Subject Headings

Adult; Blood Pressure; Body Mass Index; Cardiovascular Diseases; Cholesterol; Developed Countries; Developing Countries; Diabetes Mellitus; Ethnicity; Glycated Hemoglobin; Glycemic Control; Health Disparate Minority and Vulnerable Populations; Humans; Latin America; Lipoproteins, LDL; Minority Groups; Randomized Controlled Trials as Topic; United States; Global Health

SIMILAR REVIEWS

Check for similar records already in PROSPERO

PROSPERO identified a number of existing PROSPERO records that were similar to this one (last check made on 7 December 2025). These are shown below along with the reasons given by that the review team for the reviews being different and/or proceeding.

- Comparative effects of community-based non-communicable disease interventions in low- and middle-income countries of Asia: a systematic review and network meta-analysis [published 24 December 2020] [CRD42020221921]. The review was judged **not to be similar**
- Impact of Community-Based Interventions on Mental Health Outcomes in Low-Income and Middle-Income Countries (LMICs): A Systematic Review [published 6 April 2025] [CRD420251020594]. The review was judged **not to be similar**
- Impact of Community Health Worker interventions on glycemic control in Type 2 Diabetes care: a systematic review and meta-analysis [published 18 February 2024] [CRD42024498110]. The review was judged **not to be similar**

PROSPERO version history

- Version 1.0, published 08 Dec 2025

Disclaimer

The content of this record displays the information provided by the review team. PROSPERO does not peer review registration records or endorse their content.

PROSPERO accepts and posts the information provided in good faith; responsibility for record content rests with the review team. The guarantor for this record has affirmed that the information provided is truthful and that they understand that deliberate provision of inaccurate information may be construed as scientific misconduct.

PROSPERO does not accept any liability for the content provided in this record or for its use. Readers use the information provided in this record at their own risk.

Any enquiries about the record should be referred to the named review contact
