# Supplementary material for: Effects of community health worker–led interventions on cardiometabolic outcomes in vulnerable populations: A systematic review and meta-analysis
Source: PLOS Glob Public Health. 2026 Aug 26;6(8):e0007191. doi: 10.1371/journal.pgph.0007191 (PMC13514067; doi:10.1371/journal.pgph.0007191)
Supplement: Supplementary file 2 [file pgph.0007191.s004.pdf]

S4_File. Characteristics of CHW-led Intervention Delivery Across Included Studies

This table summarizes the delivery format, total number of participant contacts, intervention duration, and average contact frequency for each of the 30 randomized controlled trials included in this systematic review and meta-analysis.

Study	Delivery format	Total contacts	Duration	Contact frequency (per month)
Rothschild 2014	Individual (home visits)	36	24 mo	1.5
Philis-Tsimikas 2011	Group (classes + support groups)	~14	~10 mo	Tapering (~4→1)
Allen 2011	Individual (NP/CHW team)	Not quantified	12 mo	Not quantified
Nguyen 2024	Mixed (CHW + home BP + storytelling)	Not quantified	12 mo	Not quantified
O'Brien 2017	Group (sessions)	24	12 mo	2.0
Thapa 2023	Individual (home visits)	3	12 mo	0.25
Spencer 2011	Group (DSME)	Not quantified	6 mo	Not quantified
Teshome 2024	Individual (home visits)	4	9 mo	0.44
Tang 2014	Remote (phone)	12	12 mo	1.0
Ursua 2018	Mixed (workshops + visits + calls)	~16	4 mo	4.0
Tian 2015	Remote (app-guided)	12	12 mo	1.0
Vargas 2024	Individual (sessions)	16	3 mo	5.33
Balcazar 2010	Group (classes)	8	4 mo	2.0
Carrasquillo 2017	Mixed (visits + calls + groups)	~36	12 mo	3.0
Bhattarai 2024	Individual (home visits)	6	4-8 mo	Not quantified
Chen 2025	Remote (digital/app)	Daily	3 mo	Not converted
Pérez-Escamilla 2015	Individual (home visits)	17	12 mo	1.4
Fakiri 2008	Individual (clinic visits)	4	12 mo	0.33
Gyawali 2021	Individual (home visits)	3	12 mo	0.25
He 2017	Mixed (visits + texts)	~12	18 mo	0.67
Hickey 2025	Remote (telehealth)	Not quantified	24–48 wk	Not quantified
Islam 2023	Mixed (group + coaching)	≥5	6 mo	≥0.83
Joshi 2019	Individual (household visits)	6	12 mo	0.5
Jafar 2009	Individual (home visits)	8	24 mo	0.33
Kangovi 2017	Individual (individualized)	Weekly	6 mo	4.3
Khetan 2019	Individual (home visits)	Not quantified	24 mo	Not quantified
Lujan 2007	Mixed (classes + calls)	~16	~6 mo	2.7
Koniak-Griffin 2015	Mixed (classes + visits + calls)	16	6 mo	2.7
Nelson 2017	Individual (home visits)	4 (+1 optional)	12 mo	0.33
Mbuthia 2024	Individual (home visits)	6	6 mo	1.0
