# Supplementary material for: Effects of community health worker–led interventions on cardiometabolic outcomes in vulnerable populations: A systematic review and meta-analysis
Source: PLOS Glob Public Health. 2026 Aug 26;6(8):e0007191. doi: 10.1371/journal.pgph.0007191 (PMC13514067; doi:10.1371/journal.pgph.0007191)
Supplement: Supplementary file 3 [file pgph.0007191.s002.docx]

**S2 File. Search Strategy**

*“Can Community-Based Health Worker Interventions Improve Cardiometabolic Outcomes in Vulnerable Populations? A Systematic Review and Meta-Analysis”*

# Databases and Search Period

The following databases were searched for original articles from January 1, 2000 to December 20, 2025: PubMed/MEDLINE, Scopus, Embase, the Cochrane Library (CENTRAL), and ClinicalTrials.gov.

Only randomized controlled trials (RCTs) evaluating community health worker (CHW)-led interventions on cardiometabolic outcomes were considered eligible for inclusion.

# Summary of Records Retrieved

| **Database** | **Date Searched** | **Records Retrieved** |
| --- | --- | --- |
| PubMed/MEDLINE | Jan 1, 2000 – Dec 20, 2025 | 121 |
| Cochrane CENTRAL | Jan 1, 2000 – Dec 20, 2025 | 363 |
| Scopus | Jan 1, 2000 – Dec 20, 2025 | 298 |
| Embase | Jan 1, 2000 – Dec 20, 2025 | 241 |
| ClinicalTrials.gov | Jan 1, 2000 – Dec 20, 2025 | 37 |
| **Total** |  | **1,060** |

Total records retrieved across all five databases (n = 1,060) is consistent with the number reported in the PRISMA flow diagram (Fig 1).

# MeSH Terms and Keywords

Hypertension; Blood Pressure; Diabetes Mellitus; HbA1c; Body Mass Index; Cholesterol; Cardiometabolic Diseases; Community Health Workers; CHW-led Intervention; Health Promotion; Health Education; Patient Compliance; Medication Adherence; Lifestyle Modification; Randomized Controlled Trial.

# Database-Specific Search Strategies

## PubMed/MEDLINE (Boolean Format)

("Hypertension"[Mesh] OR "Blood Pressure"[Mesh] OR "systolic blood pressure"[tiab] OR "diastolic blood pressure"[tiab] OR "Diabetes Mellitus"[Mesh] OR "HbA1c"[tiab] OR "Body Mass Index"[Mesh] OR BMI[tiab] OR "Cholesterol"[Mesh])

AND

("Community Health Workers"[Mesh] OR "community-based health worker*"[tiab] OR "lay health worker*"[tiab] OR "promotores"[tiab] OR "community nurse"[tiab] OR "outreach worker*"[tiab] OR "peer educator*"[tiab])

AND

("Randomized Controlled Trial"[Publication Type] OR randomized[tiab] OR randomised[tiab] OR RCT[tiab] OR "cluster randomized"[tiab])

Date searched: January 1, 2000 – December 20, 2025. Records retrieved: 121.

## Cochrane CENTRAL

```
#1 [mh Hypertension] OR [mh "Blood Pressure"] OR [mh "Diabetes Mellitus"] OR (HbA1c):ti,ab,kw OR [mh "Body Mass Index"] OR (BMI):ti,ab,kw OR [mh Cholesterol]
#2 [mh "Community Health Workers"] OR (community-based health worker* OR lay health worker* OR promotor* OR community nurse* OR outreach worker* OR peer educator*):ti,ab,kw
#3 #1 AND #2
```

Date searched: January 1, 2000 – December 20, 2025. Records retrieved: 363.

## Scopus

TITLE-ABS-KEY("hypertension" OR "blood pressure" OR "diabetes mellitus" OR "HbA1c" OR "body mass index" OR BMI OR cholesterol)

AND

TITLE-ABS-KEY("community health worker*" OR "community-based health worker*" OR "lay health worker*" OR promotor* OR "community nurse*" OR "outreach worker*" OR "peer educator*")

AND

TITLE-ABS-KEY(randomi?ed OR RCT OR "cluster randomi?ed" OR "controlled trial")

Date searched: January 1, 2000 – December 20, 2025. Records retrieved: 298.

## Embase (Ovid or Embase.com syntax)

('hypertension'/exp OR 'blood pressure'/exp OR 'diabetes mellitus'/exp OR 'hemoglobin A1c'/exp OR 'body mass'/exp OR 'cholesterol'/exp)

AND

('community care'/exp OR 'community health worker*':ti,ab OR 'lay health worker*':ti,ab OR promotor*:ti,ab OR 'community nurse*':ti,ab OR 'outreach worker*':ti,ab OR 'peer educator*':ti,ab)

AND

('randomized controlled trial'/exp OR randomi?ed:ti,ab OR rct:ti,ab OR 'cluster randomi?ed':ti,ab)

Date searched: January 1, 2000 – December 20, 2025. Records retrieved: 241.

## ClinicalTrials.gov

Condition: hypertension OR diabetes OR obesity OR dyslipidemia

Intervention: community health worker OR lay health worker OR promotora OR peer educator OR outreach worker

Study type: Interventional (RCT)

Date searched: January 1, 2000 – December 20, 2025. Records retrieved: 37.

Note: Search strings were adapted to each database's native syntax and controlled vocabulary (e.g., Emtree for Embase, CENTRAL's own field tags for Cochrane). Equivalence across databases refers to conceptual coverage of the same population, intervention, and study-design terms, not literal string identity.

# Deduplication Plan

All citations retrieved from the database searches were imported into reference management software and deduplicated using both automated and manual screening procedures prior to title and abstract review.

# Inclusion Criteria

**Population**

Adults aged ≥18 years with hypertension, diabetes mellitus, obesity, dyslipidemia, or other cardiometabolic risk factors. Vulnerable populations, including individuals residing in low- and middle-income countries (LMICs), racial or ethnic minority populations in high-income countries (HICs), rural or underserved communities, or populations with limited access to healthcare.

**Intervention**

CHW-led interventions delivered by non-physician health workers, including lifestyle counseling, health education, medication adherence support, home blood pressure monitoring, diabetes education, health coaching, or facilitated linkage to primary care services.

**Study Design**

Randomized controlled trials (individual or cluster-randomized).

**Outcomes**

Systolic blood pressure (SBP); diastolic blood pressure (DBP); glycated hemoglobin (HbA1c); body mass index (BMI); low-density lipoprotein cholesterol (LDL-C).

**Language**

Studies published in English.

# Exclusion Criteria

**Population**

Pediatric populations (<18 years). Pregnancy-only cohorts.

**Intervention**

Physician-only interventions. Clinic-only nurse or pharmacist interventions without a community-based or outreach component.

**Study Design**

Observational studies, quasi-experimental designs, and pre-post studies without a control group. Reviews, protocols, editorials, commentaries, conference abstracts, and non-original research.

**Outcomes**

Studies not reporting at least one of the prespecified cardiometabolic outcomes (SBP, DBP, HbA1c, BMI, or LDL-C).
