# Supplementary material for: Effects of community health worker–led interventions on cardiometabolic outcomes in vulnerable populations: A systematic review and meta-analysis
Source: PLOS Glob Public Health. 2026 Aug 26;6(8):e0007191. doi: 10.1371/journal.pgph.0007191 (PMC13514067; doi:10.1371/journal.pgph.0007191)
Supplement: Supplementary file 11 [file pgph.0007191.s025.docx]

**Response to Reviewers**

**Manuscript PGPH-D-26-00622R3**

Effects of community health worker–led interventions on cardiometabolic outcomes in vulnerable populations: A systematic review and meta-analysis

PLOS Global Public Health

We thank the Academic Editor and both reviewers for their continued careful evaluation of our manuscript. We are encouraged that Reviewer #1 found the manuscript “considerably stronger than the previous version” and that Reviewer #2 indicated no further comments. Below we address each of Reviewer #1’s remaining points in turn. All changes are also reflected in the accompanying track-changes manuscript file.

**Response to Editor – Journal Requirements**

***Reviewer Comment:*** *1. If the reviewer comments include a recommendation to cite specific previously published works, please review and evaluate these publications to determine whether they are relevant and should be cited.*

**Response:** Reviewer #1 did not recommend any specific additional works to cite in this round; no action was required beyond the reference-verification changes described in response to Comment 5 below.

***Reviewer Comment:*** *2. Please review your reference list to ensure that it is complete and correct, including verification of any retracted articles.*

**Response:** We have re-reviewed the full reference list. No cited work has been retracted. All references have been re-verified against their publisher records, and one incomplete citation has been corrected (see response to Comment 5 below).

**Response to Reviewer #1**

***Reviewer Comment:*** *1. Persistent Overinterpretation of LMIC and Regional Subgroup Findings – Although the authors have moderated their language, residual overinterpretation remains. The Discussion still states: “Several mechanisms may explain the stronger blood pressure reductions observed in LMICs and certain geographic regions.” However, earlier responses acknowledge that region meta-regression was not significant and income meta-regression was not significant. The current text risks implying causal explanations for subgroup differences that were not statistically confirmed. Recommendation: Revise wording to emphasize that these explanations remain speculative and exploratory.*

**Response:** We agree and have revised this paragraph (Discussion, Section 4.1) to explicitly frame the proposed explanations as speculative and hypothesis-generating rather than confirmed mechanisms. The paragraph now opens: “Because formal meta-regression did not identify region or income classification as statistically significant effect modifiers, the following explanations are speculative and hypothesis-generating rather than confirmed mechanisms.” We also softened the causal language throughout (“may explain” → “could contribute to,” “allowing” → “could allow,” “enhance” → “could enhance,” “leading to” → “potentially yielding”), and added a closing sentence stating that these explanations “warrant direct testing in future studies designed specifically to evaluate effect modification by setting.”

***Reviewer Comment:*** *2. Contradictory Interpretation of Heterogeneity – The manuscript still contains language suggesting that heterogeneity should not necessarily be viewed as a limitation: “heterogeneity should not be viewed as a limitation that undermines effectiveness.” This statement is problematic. While contextual heterogeneity is expected in complex interventions, I² values of 96–99% remain a serious limitation to confidence in pooled effects. Recommendation: Reframe this paragraph more cautiously to avoid minimizing the implications of extreme heterogeneity.*

**Response:** We agree this sentence read as minimizing a genuine limitation. The closing sentence of Section 4.1 has been revised to: “From a policy perspective, this pattern is consistent with the intervention’s effects being context-dependent and potentially optimizable through local adaptation; nonetheless, the magnitude of unexplained heterogeneity (I² 96–99%) remains a substantive limitation that constrains confidence in the pooled effect estimates and should be weighed accordingly when interpreting these findings.” This retains the observation that heterogeneity may reflect contextual variability while explicitly restating, rather than downplaying, the limitation it poses to pooled-estimate confidence, consistent with the treatment already given in Section 4.2 (Limitations).

***Reviewer Comment:*** *3. Abstract Certainty Ratings Appear Inconsistent – The tracked-changes version indicates “lowmoderate” for SBP and DBP and “moderate” for BMI. Meanwhile Table 3 reports SBP = Low, DBP = Low, BMI = Moderate, HbA1c = Very low, LDL-C = Low. Recommendation: Carefully proofread the abstract and ensure consistency between abstract, results, and Table 3.*

**Response:** We have carefully re-checked this. In the clean manuscript file, the Abstract already states GRADE certainty as “low for systolic and diastolic blood pressure, moderate for BMI, very low for HbA1c, and low for LDL-cholesterol,” which matches Table 3 exactly. The “lowmoderate” text the reviewer observed was an artifact specific to the previous round’s track-changes redline formatting and was not present in the manuscript text itself. We have regenerated the track-changes file for this round from the correct, consistent clean manuscript to prevent any recurrence of this rendering issue, and we thank the reviewer for flagging it so we could confirm the underlying text was sound.

***Reviewer Comment:*** *4. Some Residual Editorial Artifacts Remain – Several sections appear to retain tracked-change artifacts or duplicated text, for example: “CHW-ledCommunity-based interventions”; “improved systolic blood pressure … However” formatting inconsistencies; duplicated certainty language in Section 3.3. These likely arose during revision and should be corrected before production.*

**Response:** We have carefully re-examined the manuscript and the track-changes file for artifacts of this kind. We could not locate a “CHW-ledCommunity-based” merge in the submitted files, and confirmed the clean manuscript contains no residual tracked-change markup; we believe both of these, along with the stray “However,” noted after the systolic blood pressure result, were artifacts of the previous round’s redline rendering rather than the underlying text, and the newly regenerated track-changes file for this round has been checked to ensure it does not reproduce them. We have, however, identified and corrected a genuine instance of duplicated certainty language in Section 3.3 (GRADE certainty of evidence), where the closing sentence restated the same certainty ratings already given earlier in the paragraph. The closing sentence now reads: “Taken together, these ratings indicate that current evidence for CHW-led interventions warrants cautious interpretation pending higher-quality primary trials, particularly for blood pressure and glycemic outcomes,” removing the redundant restatement while preserving the paragraph’s conclusion.

***Reviewer Comment:*** *5. Reference Verification – Most citation concerns have been addressed. However, several references remain future-dated (2025–2026). If accepted, the editorial office should ensure all references are formally published, citation details are accurate, and prepublication references are properly identified.*

**Response:** We have individually re-verified every 2025–2026-dated reference (References 2, 3, 36, 41, 56, and 57) against publisher records. All are formally published (not in-press or preprint) with complete volume, issue, and page or article-number details, with one exception that we have now corrected: Reference 57 (Tesema et al., “The case for community health workers in high-income countries”) was missing volume, page, and DOI information. We have updated it to: “Tesema AG, Osório D, Joshi R. The case for community health workers in high-income countries. Lancet Prim Care. 2026;2(1):100068. doi:10.1016/j.lanprc.2025.100068,” verified directly against the publisher’s record.

**Minor Comments**

***Reviewer Comment:*** *Methods – The manuscript now reports that 12/30 studies were low risk, 18/30 had some concerns, none were high risk. This addition is valuable and should be retained in the main text.*

**Response:** Thank you, this has been retained in the main text (Section 3.1) without change.

***Reviewer Comment:*** *Intervention Characterization – The new supplementary intervention table is helpful. Consider briefly summarizing the range of intervention durations and contact frequencies within the Results section.*

**Response:** We have added a brief summary sentence to Section 3.1 immediately before the reference to S4 File: “Intervention duration ranged from 6 to 60 months (median 12 months) across included trials, with substantial additional variation in contact frequency and delivery format.” Full study-by-study detail remains in S4 File.

***Reviewer Comment:*** *Conclusion – The revised conclusion appropriately states that findings are “hypothesis-generating rather than practice-changing.” This is an important improvement and should remain.*

**Response:** Thank you, this language has been retained without change.

**Response to Reviewer #2**

Reviewer #2 indicated that all previous comments have been addressed and provided no further comments in this round. We thank Reviewer #2 for their earlier guidance, which substantially improved the manuscript.

We believe these revisions fully address the remaining points raised and thank the Editor and both reviewers again for their continued guidance in strengthening the manuscript.
