# Supplementary material for: Effects of community health worker–led interventions on cardiometabolic outcomes in vulnerable populations: A systematic review and meta-analysis
Source: PLOS Glob Public Health. 2026 Aug 26;6(8):e0007191. doi: 10.1371/journal.pgph.0007191 (PMC13514067; doi:10.1371/journal.pgph.0007191)
Supplement: Supplementary file 12 [file pgph.0007191.s018.docx]

The authors have conducted and analyzed a systematic review and meta-analysis on current global major health crises, ‘*Effects of community health worker–led interventions on cardiometabolic outcomes in vulnerable populations: A systematic review and meta-analysis*’. However, they need to address the following issues.

1. There is a study Jeet et al. “*Jeet G, Thakur JS, Prinja S, Singh M (2017) Community health workers for non-communicable diseases prevention and control in developing countries: Evidence and implications. PLOS ONE 12(7): e0180640.* [*https://doi.org/10.1371/journal.pone.0180640*](https://doi.org/10.1371/journal.pone.0180640)” assessed and pooled the following cardiometabolic outcomes such as, Systolic Blood Pressure, Diastolic Blood Pressure, Glycated Haemoglobin/HbA1c, Weight and Body Mass Index (BMI), Total Cholesterol. You have to provide strong justification that your study adds beyond Jeet et al., as well as cite this article and discuss it in relation to your findings.
2. Line 110: in Figure 1, Specify the number of studies you obtained from each database instead of simply writing the total number of studies. The number of reports of included studies was not reported. You might have pooled one report from some studies and more than one outcome report in others. Don’t compromise the PRISMA flow diagram.
3. Line 123-125, The inclusion criteria stated that they were about underserved populations. What is your criterion for determining whether the primary study was conducted on an underserved population or not?
4. Line 155-157, How does leave-one-out detect the source of heterogeneity? Is it scientifically sound to exclude studies using leave-one-out until the heterogeneity is removed? Leave-one-out is a sensitivity test to assess how robust the pooled findings are—how the pooled result changes in magnitude and heterogeneity—but not to identify the source of heterogeneity.
5. Line 191 (table 2), Put the number of studies pooled for each category. A minimum of 10 studies is required to conduct subgroup analysis, meta-regression, publication bias, and trim-and-fill analysis. <https://www.cochrane.org/authors/handbooks-and-manuals/handbook/current/chapter-10>
6. Line 191 (table 2), You have conducted sensitivity analysis before striving to capture the source of heterogeneity. Sensitivity analysis is the last step after you have explored the possible sources of heterogeneity through subgroup analysis and meta-regression.
7. line 206(table3), certainty of evidence should be put as individual studies not as a group.
8. Line 217, the 95% CI interval is enough to see the significance, remove the p-value and replace with I-squared value instead. Do the same for the rest of the results.
9. There is variability between sample sizes. However, I haven’t seen meta-regression on sample size to account for the heterogeneity.
