# Supplementary material for: Effects of community health worker–led interventions on cardiometabolic outcomes in vulnerable populations: A systematic review and meta-analysis
Source: PLOS Glob Public Health. 2026 Aug 26;6(8):e0007191. doi: 10.1371/journal.pgph.0007191 (PMC13514067; doi:10.1371/journal.pgph.0007191)
Supplement: Supplementary file 13 [file pgph.0007191.s020.docx]

Th authors have addressed the previous comments well. Here are additional comments that needs to be addressed.

1. Line 98 states that high-income countries were also included in this study; however, the inclusion criteria in Line 126 specify that eligible studies specifically involved vulnerable or underserved populations. This is also reflected in the results section (Lines 191–205). Please be transparent about whether high-income countries were included in all search and inclusion processes unless stick to LMIC.
2. Use a consistent referencing style throughout the document. In most sections, square brackets [] are used, whereas in Line 149, parentheses () are used. Please ensure a uniform citation format is maintained consistently across the entire document.
3. Lines 149–151: Does the revised RoB-2 assess blinding and loss to follow-up? Please refer to the Cochrane Library, as these aspects have been updated in the revised version.
4. Table 2: The value (~99%) should be presented in two digits and the tilde (~) should be removed.
5. Lines 459–460: Where is the pooled effect size supporting the statement that “Community health workers are representatives of cultural diversity and represent a scalable and culturally effective approach”? Although this may be true, it is not supported by the pooled findings in this analysis. Please ensure the discussion and conclusion remains strictly aligned with the reported results.
