# Supplementary material for: Effects of community health worker–led interventions on cardiometabolic outcomes in vulnerable populations: A systematic review and meta-analysis
Source: PLOS Glob Public Health. 2026 Aug 26;6(8):e0007191. doi: 10.1371/journal.pgph.0007191 (PMC13514067; doi:10.1371/journal.pgph.0007191)
Supplement: Supplementary file 14 [file pgph.0007191.s021.docx]

**Response to Reviewers**

**Manuscript ID:** PGPH-D-26-00622

**Title:** Effects of Community Health Worker–Led Interventions on Cardiometabolic Outcomes in Vulnerable Populations: A Systematic Review and Meta-Analysis

We thank the Reviewers and Editors for their thoughtful and constructive critique of our manuscript. The comments have substantially strengthened the methodological rigor and reporting quality of this work. We have addressed each comment in full below. All changes are indicated in the revised manuscript. Reviewer comments are reproduced in italics, followed by our responses.

**Comment 1:** *There is a study Jeet et al. (2017) that assessed and pooled cardiometabolic outcomes including SBP, DBP, HbA1c, weight, BMI, and total cholesterol. You have to provide strong justification that your study adds beyond Jeet et al., as well as cite this article and discuss it in relation to your findings.*

**Response:** We thank the reviewer for drawing attention to this important prior work. Jeet et al. (2017) has now been cited and discussed in both the Introduction and Discussion sections. Although Jeet et al. examined CHW interventions for non-communicable disease prevention across diverse cardiometabolic outcomes, their review was restricted to LMICs and predates a substantial body of recent trial evidence. The present review extends Jeet et al. (2017) by incorporating newer evidence, including both LMIC and HIC settings to enable direct comparative subgroup analysis by national income classification, and synthesizing outcomes across the full spectrum of CMDs — hypertension, obesity, dyslipidemia, and diabetes — in vulnerable populations globally.

In the Discussion, we now explicitly contextualize our findings against Jeet et al.: Anand et al.'s findings showed that CHWs' task-sharing interventions reduced systolic blood pressure by 3.7 mmHg, whereas Jeet et al. demonstrated a reduction of 4.80 mmHg in LMICs — consistent with our pooled LMIC estimate of −5.09 mmHg, reflecting the expanding evidence base and the inclusion of more recent, larger trials. The Jeet et al. reference has been added to the reference list as reference 14.

**Comment 2:** *In Figure 1, specify the number of studies obtained from each database instead of simply writing the total number of studies.*

**Response:** We thank the reviewer for this observation. The PRISMA flow diagram has been updated to reflect per-database record counts in the identification box as follows: PubMed/MEDLINE (n = 121), Cochrane Library (n = 363), Scopus (n = 298), Embase (n = 241), and ClinicalTrials.gov (n = 37), yielding a combined total of 1,060 records prior to deduplication. The revised Figure 1 is included with this submission.

**Comment 3:** *The inclusion criteria stated that they were about underserved populations. What is your criterion for determining whether the primary study was conducted on an underserved population or not?*

**Response:** We thank the reviewer for this important clarification request. An operational definition of vulnerable or underserved populations has been added to the Methods section (Section 2.2) immediately following the inclusion criteria paragraph. Eligible populations were defined as those meeting at least one of five prespecified criteria as reported by primary study authors: (1) residence in a low- or middle-income country per World Bank classification; (2) self-identified racial or ethnic minority status; (3) low socioeconomic status defined by income below the federal poverty level or equivalent national threshold; (4) residence in a rural or geographically isolated community with documented barriers to healthcare access; or (5) documentation of limited healthcare access, low health literacy, or structural socioeconomic marginalization as described by study authors.

**Comment 4:** *How does leave-one-out detect the source of heterogeneity? Is it scientifically sound to exclude studies using leave-one-out until the heterogeneity is removed? Leave-one-out is a sensitivity test to assess how robust the pooled findings are, not to identify the source of heterogeneity.*

**Response:** The reviewer is correct, and we thank them for this methodological clarification. The original framing of leave-one-out as a heterogeneity source-identification tool was inaccurate. We have revised the Methods section (Section 2.4) and all relevant Results sections to accurately characterize leave-one-out as a sensitivity procedure assessing the robustness of the pooled estimate and evaluating whether any single study disproportionately influences the overall result. All language implying that leave-one-out was used to identify or remove sources of heterogeneity has been removed throughout the manuscript. Heterogeneity was instead explored through prespecified subgroup analyses and mixed-effects meta-regression, with leave-one-out serving as a confirmatory robustness check.

**Comment 5:** *Put the number of studies pooled for each category. A minimum of 10 studies is required to conduct subgroup analysis, meta-regression, publication bias, and trim-and-fill analysis.*

**Response:** We thank the reviewer for this important observation. We acknowledge that the Africa subgroup comprised only three studies (Hickey 2025, Mbuthia 2024, Teshome 2024), which falls below the minimum of 10 studies recommended by the Cochrane Handbook for formal subgroup analysis, meta-regression, and publication bias testing. Accordingly, we have removed formal Egger's test and trim-and-fill results for the Africa subgroup and have explicitly recharacterized these findings as exploratory in both the Results text and Table 2. A qualifying statement has been added to Section 3.4.1 noting that formal publication bias testing was restricted to analyses with ≥10 contributing studies. The number of contributing studies has been added to Table 2 for all subgroup and outcome analyses.

**Comment 6:** *You have conducted sensitivity analysis before striving to capture the source of heterogeneity. Sensitivity analysis is the last step after you have explored the possible sources of heterogeneity through subgroup analysis and meta-regression.*

**Response:** We thank the reviewer for this important point regarding analytical sequencing. The Methods section (Section 2.4) has been revised to present the correct analytical order: subgroup analyses and meta-regression are now explicitly described as the primary heterogeneity exploration procedures, followed by leave-one-out sensitivity analysis as a confirmatory robustness check. This sequencing is now consistently reflected in both the Methods and Results sections throughout the manuscript.

**Comment 7:** *Certainty of evidence should be put as individual studies not as a group.*

**Response:** We respectfully clarify that GRADE certainty of evidence assessments are, by methodological design, applied at the outcome level across the body of evidence rather than at the individual study level. This is consistent with the GRADE handbook and standard systematic review reporting practice, in which certainty ratings reflect the cumulative evidence for each outcome rather than individual trial quality. Our Table 3 is therefore correctly structured. To ensure clarity, we have added explicit GRADE certainty statements to each individual results section (Sections 3.4 through 3.8), summarizing the certainty rating and the specific domains that led to downgrading for each outcome. We hope this addresses the reviewer's underlying concern about the transparency of the certainty assessments.

**Comment 8:** *The 95% CI interval is enough to see the significance, remove the p-value and replace with I-squared value instead.*

**Response:** We thank the reviewer for this suggestion. P-values in the results text have been replaced with I² statistics throughout all results sections (Sections 3.4 through 3.8). P-values are retained only within tables where both metrics provide complementary information. This change has been applied consistently across all five outcome sections.

**Comment 9:** *There is variability between sample sizes. However, I haven't seen meta-regression on sample size to account for the heterogeneity.*

**Response:** We thank the reviewer for raising this point. Mixed-effects meta-regression incorporating sample size as a continuous moderator has been performed and reported for all five outcomes. Sample size did not emerge as a statistically significant moderator for any outcome (all p > 0.05), indicating that between-study heterogeneity was not explained by study size. These findings have been incorporated into each results section (Sections 3.4 through 3.8) and are consistent with the interpretation that observed heterogeneity reflects contextual variability in intervention delivery, population characteristics, and healthcare infrastructure rather than a systematic small-study effect.

**Summary of Changes**

- Jeet et al. (2017) cited and discussed in Introduction and Discussion; added as reference 14
- PRISMA flow diagram updated with per-database record counts
- Operational definition of vulnerable/underserved populations added to Methods
- Leave-one-out reframed as sensitivity procedure throughout Methods and Results
- Africa subgroup (n = 3 studies) recharacterized as exploratory; formal bias tests removed
- Analytical sequence corrected to subgroup → meta-regression → sensitivity analysis
- GRADE certainty statements added to each individual results section
- P-values replaced with I² statistics in all results text
- Sample size meta-regression performed and reported for all five outcomes
- Participant count harmonized to 15,295 throughout
- HbA1c result corrected from "non-significant" to statistically significant
- Abstract updated to reflect I², GRADE certainty ratings, and subgroup findings
- Conclusion updated with GRADE language

We believe these revisions have substantially strengthened the manuscript and have addressed all reviewer concerns comprehensively. We are grateful for the opportunity to improve this work and hope the revised manuscript is now suitable for publication in PLOS Global Public Health.

Sincerely,

Micah Nnabuko Okwah, MD On behalf of all authors

Department of Epidemiology, Yale School of Public Health New Haven, Connecticut, USA [micah.okwah@yale.edu](mailto:micah.okwah@yale.edu)
