# Supplementary material for: Effects of community health worker–led interventions on cardiometabolic outcomes in vulnerable populations: A systematic review and meta-analysis
Source: PLOS Glob Public Health. 2026 Aug 26;6(8):e0007191. doi: 10.1371/journal.pgph.0007191 (PMC13514067; doi:10.1371/journal.pgph.0007191)
Supplement: Supplementary file 15 [file pgph.0007191.s022.docx]

The authors have addressed the previous comments; however, some conclusions remain overstated and are not supported by the findings. This issue needs to be addressed.

1. Abstract: Try to reduce abbreviations in the abstract. For variables written in abbreviation, provide the full form at least once (e.g., LMIC, HICs).

2. Data availability statement: You have generated new datasets from primary studies. Therefore, check whether your results are correctly reflected in the data availability statement.

3. S2 File 2 (Search strategy): You have included only the search strings, but you need to be transparent and provide all necessary information about the search strategy in a tabular format, including:

● The database name

● The search strings used in that database

● The date when the search was conducted

● The number of studies identified

4. “Equivalent search strategies were adapted for Scopus, Embase, Cochrane CENTRAL, and ClinicalTrials.gov.” In Supplementary File 2, how were they equivalent? Clearly present the search strategy for each database.

5. Line 113: Remove vague words such as “inception.”

6. In the Data Extraction and Quality Assessment section, you have abbreviated the roles of extractors and discrepancy handlers, but not in the Literature Search and Eligibility Criteria sections. Please maintain consistency.

7. Lines 439–452: Where in your manuscript have you assessed the link between follow-up duration and improvement in your outcome variables? Your recommendations (future directions) should be aligned with your findings.

8. Lines 461–463: Instead of using general terms like “contextual and health system factors,” be clear and specific in your conclusion. State exactly what you have found.
