# Supplementary material for: Effects of community health worker–led interventions on cardiometabolic outcomes in vulnerable populations: A systematic review and meta-analysis
Source: PLOS Glob Public Health. 2026 Aug 26;6(8):e0007191. doi: 10.1371/journal.pgph.0007191 (PMC13514067; doi:10.1371/journal.pgph.0007191)
Supplement: Supplementary file 16 [file pgph.0007191.s023.docx]

**Response to Reviewers**

**Manuscript ID:** PGPH-D-26-00622

**Title:** Effects of Community Health Worker–Led Interventions on Cardiometabolic Outcomes in Vulnerable Populations: A Systematic Review and Meta-Analysis

We thank the Reviewers and Editors for their thoughtful and constructive critique of our manuscript. The comments have substantially strengthened the methodological rigor and reporting quality of this work. We have addressed each comment in full below. All changes are indicated in the revised manuscript. Reviewer comments are reproduced in italics, followed by our responses.

**Reviewer Comment 1:**
*Line 98 states that high-income countries were also included in this study; however, the inclusion criteria in Line 126 specify that eligible studies specifically involved vulnerable or underserved populations. This is also reflected in the results section (Lines 191–205). Please be transparent about whether high-income countries were included in all search and inclusion processes, unless you stick to LMIC.*

**Response:**
We thank the reviewer for this important observation. We have clarified the eligibility criteria (see section 2.2 to explicitly state that study inclusion was based on population vulnerability rather than national income classification. We now specify that both low- and middle-income countries (LMICs) and high-income countries (HICs) were eligible, provided that the study population comprised vulnerable or underserved groups. We also clarified that the literature search was conducted without geographic restrictions. These revisions improve transparency and ensure consistency between the introduction, methods, and results sections.

**Reviewer Comment 2:**
*Use a consistent referencing style throughout the document. In most sections, square brackets [] are used, whereas in Line 149, parentheses () are used. Please ensure a uniform citation format is maintained consistently across the entire document.*

**Response:**
Thank you for identifying this inconsistency. We carefully reviewed the manuscript and standardized the citation format throughout the document. All references now follow a uniform square-bracket citation style in accordance with the journal’s formatting requirements.

**Reviewer Comment 3:**
*Lines 149–151: Does the revised RoB-2 assess blinding and loss to follow-up? Please refer to the Cochrane Library, as these aspects have been updated in the revised version.*

**Response:**
Thank you for this helpful comment. We confirm that this study used the revised Cochrane Risk of Bias 2 (RoB 2) tool, which is the most current version recommended by Cochrane for assessing risk of bias in randomized controlled trials. Upon reviewing the reviewer’s comment, we recognized that the manuscript did not clearly describe the updated RoB 2 framework and its domains. We therefore revised the Methods section to explicitly state that the revised RoB 2 tool was used and to accurately describe its five domains, including bias arising from deviations from intended interventions and bias due to missing outcome data, which encompass concerns related to blinding and loss to follow-up, respectively. In addition, Figure 2 presents the risk-of-bias assessment based on the revised Cochrane RoB 2 methodology. These revisions improve clarity and ensure consistency with current Cochrane guidance.

**Reviewer Comment 4:**
*Table 2: The value (~99%) should be presented in two digits and the tilde (~) should be removed.*

**Response:**
Thank you for pointing this out. We have corrected Table 2 by removing the tilde symbol and reporting the heterogeneity estimate using the exact value (99.1%), ensuring consistency and precision in data presentation.

**Reviewer Comment 5:**
*Lines 459–460: Where is the pooled effect size supporting the statement that “Community health workers are representatives of cultural diversity and represent a scalable and culturally effective approach”? Although this may be true, it is not supported by the pooled findings in this analysis. Please ensure the discussion and conclusion remains strictly aligned with the reported results.*

**Response:**
We appreciate this important observation. We agree that the statement was not directly supported by the pooled quantitative findings of the present meta-analysis. Accordingly, we removed the unsupported assertion regarding cultural diversity and cultural effectiveness from the discussion and conclusion. The revised conclusion now focuses exclusively on findings supported by the pooled analyses, including the observed improvements in blood pressure, glycemic control, and body mass index, and the potential role of community health worker-led interventions as components of community-based cardiovascular risk reduction programs.

We believe these revisions have substantially strengthened the manuscript and have addressed all reviewer concerns comprehensively. We are grateful for the opportunity to improve this work and hope the revised manuscript is now suitable for publication in PLOS Global Public Health.

Sincerely,

Micah Nnabuko Okwah, MD On behalf of all authors

Department of Epidemiology, Yale School of Public Health New Haven, Connecticut, USA [micah.okwah@yale.edu](mailto:micah.okwah@yale.edu)
