# Supplementary material for: Effects of community health worker–led interventions on cardiometabolic outcomes in vulnerable populations: A systematic review and meta-analysis
Source: PLOS Glob Public Health. 2026 Aug 26;6(8):e0007191. doi: 10.1371/journal.pgph.0007191 (PMC13514067; doi:10.1371/journal.pgph.0007191)
Supplement: Supplementary file 17 [file pgph.0007191.s024.docx]

**Response to Reviewers**

**Manuscript PGPH-D-26-00622R2**

Effects of community health worker–led interventions on cardiometabolic outcomes in vulnerable populations: A systematic review and meta-analysis

PLOS Global Public Health

We thank the Academic Editor and both reviewers for their thorough and constructive re-review of our manuscript. We have carefully addressed every point raised. Below we reproduce each comment in full and describe the corresponding revision, with manuscript line/section references reflecting the newly revised version. All changes are also reflected in the track-changes manuscript file accompanying this letter.

# **Response to Reviewer #1**

## **Major Concerns**

***Reviewer Comment:*** *1. Extreme Heterogeneity Undermines Pooled Estimates - I² values approaching 99% across nearly all outcomes raise questions about whether pooled summary estimates are meaningful. The manuscript should present prediction intervals, conduct more extensive moderator analyses, and discuss whether pooling was appropriate at all.*

***Response:*** We agree that the extremely high heterogeneity required more transparent quantification and exploration. We have made the following changes:

- Added 95% prediction intervals for all five pooled outcomes (SBP, DBP, BMI, LDL-C, HbA1c) immediately following each pooled effect estimate in Section 3.4-3.8, and summarized them in the Abstract and Discussion. All five prediction intervals cross the null, which we now state explicitly as evidence of substantial uncertainty in the expected effect at a new site.
- Conducted additional meta-regression testing delivery format (individual, group-based, mixed, remote/digital), intervention duration, and their interaction with national income classification as moderators for every outcome (Sections 3.4-3.8). For BMI, delivery format and intervention duration were significant moderators, together explaining 70-78% of between-study heterogeneity (both p<0.01). For SBP, DBP, LDL-C, and HbA1c, none of the tested moderators reached significance (all omnibus p>0.1), meaning heterogeneity for those outcomes remains largely unexplained.
- Revised the Discussion to explicitly state that the substantial unexplained heterogeneity (I² 96-99% across outcomes) and null-crossing prediction intervals limit the generalizability of pooled estimates to any single setting, rather than presenting the pooled effects as broadly generalizable (see response to Minor Concern 3 below).

***Reviewer Comment:*** *2. Inappropriate GRADE Assessment of Inconsistency - The manuscript states inconsistency was not downgraded despite substantial heterogeneity; given I² values approaching 99%, the rationale for retaining “moderate certainty” evidence appears weak.*

***Response:*** We agree with this assessment and have revised our GRADE ratings accordingly. Because the moderator analyses described above found that tested moderators did not explain the heterogeneity in SBP, DBP, or HbA1c, we have downgraded these outcomes for inconsistency: SBP and DBP are now rated as low certainty (downgraded from moderate), and HbA1c as very low certainty (downgraded from moderate). BMI was not downgraded for inconsistency, because delivery format and intervention duration jointly explained 70-78% of its between-study heterogeneity (p<0.01) an explained, rather than unexplained, source of variability. LDL-C remains rated low certainty. Table 3 and the accompanying narrative in Section 3.3, along with each outcome-specific GRADE sentence in Sections 3.4-3.8, have been revised to reflect these ratings and to state the specific rationale (risk of bias, inconsistency, indirectness, imprecision, and publication bias) for each downgrade.

***Reviewer Comment:*** *3. Broad and Potentially Problematic Definition of Vulnerability – The review combines U.S. Latino populations, Filipino Americans, rural Nepalese populations, low-income Argentinians, and African rural populations into a single pooled estimate. Provide a stronger theoretical justification for combining these populations.*

***Response:*** Our eligibility criteria (Section 2.2) already operationalize “vulnerable or underserved” populations using five author-reported criteria (LMIC residence, racial/ethnic minority status, low socioeconomic status, rural/geographically isolated residence, or explicitly documented limited healthcare access/health literacy/structural marginalization), reflecting a social-determinants-of-health framework in which the shared feature across these groups is structurally constrained access to routine preventive cardiometabolic care rather than a shared geography or income level. We have expanded this justification in Section 2.2 to state explicitly why groups differing in geographic and cultural context were considered together: CHW-led task-shifting models are proposed as a response to a common access barrier across these settings, and our pre-specified subgroup and meta-regression analyses by region and income classification (Sections 3.4–3.8) are the mechanism by which we test rather than assume whether pooling across these groups is empirically justified.

***Reviewer Comment:*** *4. Subgroup Conclusions Exceed Statistical Evidence – The manuscript emphasizes greater effectiveness in LMICs and Africa despite non-significant region (p=0.144) and income (p=0.18) meta-regressions, risking ecological overinterpretation.*

***Response:*** We have tempered our interpretation throughout the manuscript. The Abstract conclusion now explicitly notes that meta-regression did not identify specific intervention design features (contact frequency, duration, delivery format) as significant drivers of effectiveness. The manuscript Conclusion has been revised to attribute observed subgroup variability to national income classification and regional health system context rather than intervention design, while explicitly labeling these subgroup findings as hypothesis-generating rather than practice-changing, given the predominantly low-to-very-low certainty evidence across outcomes. We have also revised the Discussion's generalizability statement (see Minor Concern 3) to foreground the heterogeneity and prediction-interval uncertainty alongside the subgroup findings.

***Reviewer Comment:*** *5. Publication Bias Interpretation Is Inconsistent; The discussion largely dismisses trim-and-fill attenuation to non-significance for several outcomes despite reporting it.*

***Response:*** We have revised the Abstract and Results to state plainly that trim-and-fill sensitivity analyses attenuated the BMI and HbA1c effects to non-significance, framing this as an indication of possible publication bias rather than downplaying it. Table 3 now rates publication bias as “serious” for SBP, DBP, BMI, and HbA1c, and “not serious” for LDL-C, and the GRADE narrative (Section 3.3) and each outcome-specific GRADE sentence (Sections 3.4, 3.5, 3.8) explicitly cite trim-and-fill attenuation as contributing to the certainty rating where relevant.

***Reviewer Comment:*** *6. Clinical Significance Requires Greater Discussion; Pooled effects are statistically significant but modest (e.g., SBP: −3.72 mmHg); clinical significance for individual patients deserves more nuanced discussion.*

***Response:*** The Discussion already situates the pooled SBP reduction against trial evidence on cardiovascular outcomes (citing evidence that population-level blood pressure reductions of this magnitude are associated with meaningful reductions in cardiovascular events). We have supplemented this discussion with an explicit caveat, alongside the newly added prediction intervals, that these are average population-level effects: given intervals crossing the null for all five outcomes, the expected benefit for an individual patient at a new site is considerably less certain than the pooled mean effect alone would suggest.

***Reviewer Comment:*** *7. Search Strategy Transparency – Only a simplified example search string is provided; include complete search strings in supplementary materials and ensure reproducibility.*

***Response:*** S2 File has been rebuilt as a complete, tabular search strategy documenting, for each of the five databases (PubMed/MEDLINE, Cochrane CENTRAL, Scopus, Embase, and ClinicalTrials.gov), the full search string used, the date the search was conducted, and the number of records identified. The totals reconcile with the PRISMA flow diagram (Figure 1; n = 1,060 records identified). This directly addresses Reviewer #2's related Comments 3 and 4 below as well.

## **Minor Concerns**

***Reviewer Comment:*** *1. Terminology – The manuscript alternates between “community-based interventions,” “community health worker interventions,” and “CHW-led interventions.” These should be standardized.*

***Response:*** We have standardized terminology throughout the manuscript to “CHW-led interventions,” including in the Abstract, Introduction (Section 1), and all instances in Section headers 3.4–3.9 and the surrounding body text (e.g., variants such as “community-based health worker interventions” and “Community-Based health worker interventions”).

***Reviewer Comment:*** *2. References – Several citations supporting claims about global CMD burden appear to rely on future-dated reports (2025–2026); please verify these were publicly available at the time of manuscript preparation.*

***Response:*** We have verified both references and confirm they are legitimate, publicly available sources: reference #2 (WHO, Global report on hypertension 2025) was published in September 2025, and reference #3 (Teufel et al., Diabetes Care) was published online January 28, 2026. Both were incorporated during the revision process, which has extended into 2026; we have confirmed both were publicly accessible prior to the current submission of this revised manuscript.

***Reviewer Comment:*** *3. Discussion Overstates Generalizability – The statement that inclusion of 30 RCTs across multiple regions “strengthens the generalizability of results” should be qualified given the heterogeneity, diverse intervention models, and varied populations.*

***Response:*** This sentence has been revised to read: “While the inclusion of 30 RCTs across multiple regions provides broad geographic representation, the substantial between-study heterogeneity (I² 96–99% across outcomes) limits the generalizability of pooled effect estimates to any single setting; this is further underscored by prediction intervals that crossed the null for all five outcomes.”

***Reviewer Comment:*** *4. Intervention Characterization – A table detailing CHW training duration, supervision structure, intervention frequency, and intensity would help identify likely sources of heterogeneity.*

***Response:*** We have added a new supplementary file (S4 File: Intervention Characteristics) tabulating delivery format, contact frequency, and intervention duration across all 30 included studies, referenced in Section 3.1. Training duration and supervision structure were inconsistently reported across primary studies and could not be reliably tabulated for the majority of trials; we note this limitation directly beneath the table rather than presenting incomplete data as if it were systematically captured.

## **Specific Questions for the Authors**

***Reviewer Comment:*** *1. Why was inconsistency not downgraded in GRADE despite I² values approaching 99%?*

***Response:*** As detailed in our response to Major Concern 2 above, we have revised this: inconsistency is now downgraded for SBP, DBP, and HbA1c, whose heterogeneity was not explained by any tested moderator. It was not downgraded for BMI, where moderators explained 70-78% of between-study heterogeneity.

***Reviewer Comment:*** *2. Were prediction intervals calculated? If not, why?*

***Response:*** Prediction intervals have now been calculated and reported for all five outcomes, in the Abstract, Results (Sections 3.4-3.8), and Discussion.

***Reviewer Comment:*** *3. How were cluster-randomized trials adjusted statistically?*

***Response:*** We have added a sentence to the statistical methods (Section 2.4) clarifying that for cluster-randomized trials, effect estimates and variances were used as reported by the original trial investigators.

***Reviewer Comment:*** *4. Were intervention characteristics evaluated as moderators?*

***Response:*** Yes. Delivery format and intervention duration (and their interaction with income classification) were tested as moderators for all five outcomes via meta-regression (Sections 3.4-3.8); results are summarized in our response to Major Concern 1. The new S4 File also tabulates these characteristics at the study level.

***Reviewer Comment:*** *5. How robust are conclusions after trim-and-fill attenuation?*

***Response:*** We now state directly, in the Abstract and Results, that trim-and-fill sensitivity analyses attenuated the BMI and HbA1c effects to non-significance, and this is reflected as “serious” publication bias in the revised Table 3 for those outcomes (and for SBP and DBP). Our conclusions have been reframed as hypothesis-generating rather than definitive in light of this.

***Reviewer Comment:*** *6. What proportion of included studies were judged high risk of bias versus “some concerns”?*

***Response:*** We have added explicit proportions to the Results where risk of bias is first narrated: of the 30 included trials, 12 (40%) were judged overall low risk of bias and 18 (60%) some concerns; no trial was rated high risk. Risk of bias was most frequently elevated in domain 5 (selection of the reported result; 23/30 some concerns), while domains 2 (deviations from intended intervention) and 4 (outcome measurement) were rated low risk in all 30 trials.

# **Response to Reviewer #2**

The reviewer noted that some conclusions remain overstated and are not supported by the findings. We have addressed this concern throughout the manuscript (see in particular our responses to Reviewer #1, Major Concerns 2, 4, and 5, and Minor Concern 3 above), and address each specific point below.

***Reviewer Comment:*** *1. Abstract: Try to reduce abbreviations in the abstract. For variables written in abbreviation, provide the full form at least once (e.g., LMIC, HICs).*

***Response:*** “LMICs” and “HICs” are now spelled out in full “low- and middle-income countries (LMICs)” and “high-income countries (HICs)” at first mention in the Abstract, with the abbreviation used thereafter.

***Reviewer Comment:*** *2. Data availability statement: You have generated new datasets from primary studies. Therefore, check whether your results are correctly reflected in the data availability statement.*

***Response:*** The Data Availability Statement has been revised from “No new datasets were generated or analyzed for this study” to state that the dataset extracted and analyzed during this study (study-level effect estimates for all outcomes) is available as Supporting Information (S5 File). The extraction file has been prepared for deposit as supplementary data alongside the revised submission.

***Reviewer Comment:*** *3. S2 File 2 (Search strategy): Provide all necessary information about the search strategy in a tabular format, including the database name, the search strings used in that database, the date the search was conducted, and the number of studies identified.*

***Response:*** S2 File has been rebuilt as a single table with one row per database (PubMed/MEDLINE, Cochrane CENTRAL, Scopus, Embase, ClinicalTrials.gov), each with the complete search string, the search date, and the number of records identified, totaling 1,060 records reconciled with the PRISMA flow diagram.

***Reviewer Comment:*** *4. “Equivalent search strategies were adapted for Scopus, Embase, Cochrane CENTRAL, and ClinicalTrials.gov.” In Supplementary File 2, how were they equivalent? Clearly present the search strategy for each database.*

***Response:*** The rebuilt S2 File (see response to Comment 3) presents the full, database-specific search string for each of the five databases individually, rather than asserting equivalence, so readers can verify how the strategy was adapted to each platform's syntax.

***Reviewer Comment:*** *5. Line 113: Remove vague words such as “inception.”*

***Response:*** The search date range has been changed from “from inception to December 20, 2025” to “from January 1, 2000 to December 20, 2025,” in both the manuscript body and S2 File.

***Reviewer Comment:*** *6. In the Data Extraction and Quality Assessment section, you have abbreviated the roles of extractors and discrepancy handlers, but not in the Literature Search and Eligibility Criteria sections. Please maintain consistency.*

***Response:*** We have added reviewer initials to the Literature Search section for consistency: “Two independent authors (I.A.Y. and I.A.) conducted a thorough review… a third reviewer (M.O.) resolved the dispute,” matching the convention already used in the Data Extraction and Quality Assessment section.

***Reviewer Comment:*** *7. Lines 439-452: Where in your manuscript have you assessed the link between follow-up duration and improvement in your outcome variables? Your recommendations (future directions) should be aligned with your findings.*

***Response:*** This recommendation has been revised to align with our actual findings: “Future trials should incorporate longer follow-up to determine whether observed improvements are sustained; existing evidence within this review (e.g., attenuated BMI effects in longer-duration trials, and the COBIN trial's loss of benefit at 5-year follow-up) suggests treatment effects may not persist after intervention cessation, and meta-regression confirmed intervention duration was not associated with larger effects for SBP, DBP, HbA1c, or LDL.”

***Reviewer Comment:*** *8. Lines 461-463: Instead of using general terms like “contextual and health system factors,” be clear and specific in your conclusion. State exactly what you have found.*

***Response:*** The Conclusion has been revised to state findings specifically: “Subgroup analyses demonstrated significantly greater SBP reduction in LMICs than HICs, with the largest effects in African settings, suggesting that national income classification and regional health system context rather than specific intervention design characteristics, which were not significant moderators in meta-regression account for much of the observed variability in effectiveness. Given predominantly low to very low certainty evidence across outcomes, these findings should be interpreted as hypothesis-generating rather than practice-changing pending higher-quality primary trials.”

We believe these revisions have substantially strengthened the manuscript and thank the Editor and both reviewers again for their guidance.
