# Supplementary material for: Effects of community health worker–led interventions on cardiometabolic outcomes in vulnerable populations: A systematic review and meta-analysis
Source: PLOS Glob Public Health. 2026 Aug 26;6(8):e0007191. doi: 10.1371/journal.pgph.0007191 (PMC13514067; doi:10.1371/journal.pgph.0007191)
Supplement: Peer review history

# Peer review: Decision Letter 0

**DOI:** 10.1371/journal.pgph.0007191.r001

**Authors:** Zulkarnain Jaafar

13 May 2026

PGPH-D-26-00622

Effects of community health worker–led interventions on cardiometabolic outcomes in vulnerable populations: A systematic review and meta-analysis

PLOS Global Public Health

Dear Dr. Okwah,

Thank you for submitting your manuscript to PLOS Global Public Health. After careful consideration, we feel that it has merit but does not fully meet PLOS Global Public Health’s publication criteria as it currently stands. Therefore, we invite you to submit a revised version of the manuscript that addresses the points raised during the review process.

==============================

EDITOR: Dear Author, please make necessary corrections based on the comments provided by the reviewers.

==============================

Please submit your revised manuscript by 11th August 2026. If you will need more time than this to complete your revisions, please reply to this message or contact the journal office at globalpubhealth@plos.org. When you're ready to submit your revision, log on to https://www.editorialmanager.com/pgph/ and select the 'Submissions Needing Revision' folder to locate your manuscript file.

Please include the following items when submitting your revised manuscript:

- A letter that responds to each point raised by the editor and reviewer(s). You should upload this letter as a separate file labeled 'Response to Reviewers'.
- A marked-up copy of your manuscript that highlights changes made to the original version. You should upload this as a separate file labeled 'Revised Manuscript with Track Changes'.
- An unmarked version of your revised paper without tracked changes. You should upload this as a separate file labeled 'Manuscript'.

Guidelines for resubmitting your figure files are available below the reviewer comments at the end of this letter.

As the corresponding author, your ORCID iD is verified in the submission system and will appear in the published article. PLOS supports the use of ORCID, and we encourage all coauthors to register for an ORCID iD and use it as well. Please encourage your coauthors to verify their ORCID iD within the submission system before final acceptance, as unverified ORCID iDs will not appear in the published article. *Only*  the individual author can complete the verification step; PLOS staff *cannot*  verify ORCID iDs on behalf of authors.

We look forward to receiving your revised manuscript.

Kind regards,

Zulkarnain Jaafar

Academic Editor

PLOS Global Public Health

Journal Requirements:

If the reviewer comments include a recommendation to cite specific previously published works, please review and evaluate these publications to determine whether they are relevant and should be cited. There is no requirement to cite these works unless the editor has indicated otherwise.

Additional Editor Comments (if provided):

[Note: HTML markup is below. Please do not edit.]

Reviewers' comments:

Reviewer's Responses to Questions

**Comments to the Author**

1. Does this manuscript meet PLOS Global Public Health’s publication criteria?

Reviewer #1: Yes

Reviewer #2: Yes

**********

2. Has the statistical analysis been performed appropriately and rigorously?

Reviewer #1: N/A

Reviewer #2: No

**********

3. Have the authors made all data underlying the findings in their manuscript fully available (please refer to the Data Availability Statement at the start of the manuscript PDF file)?

The PLOS Data policy

Reviewer #1: Yes

Reviewer #2: Yes

**********

4. Is the manuscript presented in an intelligible fashion and written in standard English?

Reviewer #1: Yes

Reviewer #2: Yes

**********

Reviewer #1: Reviewer Report

Journal: PLOS Global Public Health

Manuscript ID: PGPH-D-26-00622

Title: Predictors of Exclusive Breastfeeding among Under-Five Children in Rural Ethiopia

1. Overall Assessment

This manuscript investigates predictors of exclusive breastfeeding (EBF) in rural Ethiopia using nationally representative 2019 EMDHS data and survival analysis techniques. The topic is highly relevant to global public health, particularly in low-resource settings where optimal infant feeding practices significantly influence morbidity and mortality outcomes.

The study has clear policy relevance and employs appropriate statistical techniques. However, substantial clarification and revision are required before the manuscript meets the standards of PLOS Global Public Health, particularly regarding conceptual framing, interpretation of findings, and methodological transparency.

2. Significance and Relevance to Global Public Health

The manuscript addresses:

• Maternal and child nutrition

• Social determinants of health

• Regional inequities in breastfeeding practices

• Health communication access

These themes align well with the journal’s global health mission. However, the manuscript would benefit from stronger positioning within:

• SDG 2 (Zero Hunger)

• SDG 3 (Good Health and Well-being)

• Equity-focused frameworks in global health

The discussion should more explicitly connect findings to broader global health systems strengthening and maternal-child health interventions.

3. Major Comments

3.1 Conceptual Framing of “Under-Five” Population

The title and manuscript repeatedly refer to “under-five children,” yet exclusive breastfeeding applies only to infants aged 0–6 months.

• Please clarify whether the sample includes only infants under six months or retrospectively includes all under-five children.

• If analysis focuses on EBF duration during the first six months, the title should be revised accordingly.

This conceptual imprecision may mislead readers.

3.2 Interpretation of Hazard Ratios

The manuscript reports that:

Early initiation of breastfeeding within 1 hour increases the risk of early cessation of EBF.

This finding contradicts substantial global evidence and WHO recommendations.

Possible issues:

• Misinterpretation of hazard ratios

• Incorrect reference category

• Model specification issues

The authors must:

• Re-express the hazard ratio interpretation clearly.

• Verify coding of the early initiation variable.

• Provide theoretical explanation if the finding is robust.

Without clarification, this undermines the credibility of the results.

3.3 Radio Exposure and Increased Risk of EBF Cessation

The finding that radio access increases risk of early cessation contradicts literature suggesting media exposure improves health behaviors.

The discussion currently speculates but does not sufficiently justify this counterintuitive finding.

The authors should:

• Clarify whether “radio access” measures frequency or simple ownership.

• Discuss potential confounding factors (urban influence, formula marketing exposure, etc.).

• Consider whether residual confounding or model misspecification explains the result.

3.4 Methodological Transparency

The manuscript mentions evaluating 35 predictors but does not clearly describe:

• Variable selection procedure

• Handling of multicollinearity

• Criteria for model inclusion

• Testing of proportional hazards assumption (e.g., Schoenfeld residuals)

These elements are essential for reproducibility.

Additionally:

• Provide justification for choosing Gompertz distribution as the final parametric model.

• Report model diagnostics explicitly.

3.5 Cross-Sectional Design and Causality

The DHS is cross-sectional. While survival analysis is applied, causal inference cannot be established.

The manuscript should:

• Explicitly acknowledge the limits of causal interpretation.

• Avoid causal language (e.g., “increases risk” → “is associated with increased hazard”).

4. Minor Comments

4.1 Abstract

• Replace “the vast majority (84.9%) do not exclusively breastfeed” with a neutral formulation:

“Only 15.1% of infants were exclusively breastfed.”

• Clarify time frame of EBF measurement.

4.2 Introduction

• The literature review is broad but somewhat diffuse.

• Consider tightening focus on East Africa and Ethiopia-specific policy context.

• Update citations to ensure no future-dated references are included.

4.3 Tables and Figures

• Ensure consistency in decimal places.

• Some confidence intervals appear unusually wide (e.g., HR 0.573, CI 0.446–1.737). Please verify calculations.

• Consider presenting adjusted hazard ratios only in the main table and move unadjusted results to supplementary material.

4.4 Language and Style

• Minor grammatical revisions needed throughout.

• Replace causal language with association-based phrasing.

• Ensure consistent terminology: “exclusive breastfeeding,” “EBF,” “cessation.”

4.5 Limitations Section

Currently underdeveloped. Please expand to include:

• Recall bias (maternal reporting)

• Social desirability bias

• Residual confounding

• Lack of qualitative/contextual variables

• Inability to measure breastfeeding intensity or mixed feeding patterns

5. Ethical and Reporting Considerations

• Confirm DHS ethical approval and secondary data usage permission.

• Ensure STROBE reporting checklist compliance (recommended for observational studies).

6. Strengths

• Use of nationally representative dataset

• Appropriate survival modeling framework

• Regional disaggregation of predictors

• Clear public health relevance

7. Editorial Recommendation

Major Revision Required

The study has potential to make a meaningful contribution to global maternal-child health literature. However, clarification of analytical procedures, correction of potentially misinterpreted findings, and stronger conceptual framing are necessary before the manuscript is suitable for publication.

Reviewer #2: The authors have conducted and analyzed a systematic review and meta-analysis on current global major health crises, ‘Effects of community health worker–led interventions on cardiometabolic outcomes in vulnerable populations: A systematic review and meta-analysis’. However, they need to address the following issues.

1.There is a study Jeet et al. “Jeet G, Thakur JS, Prinja S, Singh M (2017) Community health workers for non-communicable diseases prevention and control in developing countries: Evidence and implications. PLOS ONE 12(7): e0180640. https://doi.org/10.1371/journal.pone.0180640” assessed and pooled the following cardiometabolic outcomes such as, Systolic Blood Pressure, Diastolic Blood Pressure, Glycated Haemoglobin/HbA1c, Weight and Body Mass Index (BMI), Total Cholesterol. You have to provide strong justification that your study adds beyond Jeet et al., as well as cite this article and discuss it in relation to your findings.

2.Line 110: in Figure 1, Specify the number of studies you obtained from each database instead of simply writing the total number of studies. The number of reports of included studies was not reported. You might have pooled one report from some studies and more than one outcome report in others. Don’t compromise the PRISMA flow diagram.

3.Line 123-125, The inclusion criteria stated that they were about underserved populations. What is your criterion for determining whether the primary study was conducted on an underserved population or not?

4.Line 155-157, How does leave-one-out detect the source of heterogeneity? Is it scientifically sound to exclude studies using leave-one-out until the heterogeneity is removed? Leave-one-out is a sensitivity test to assess how robust the pooled findings are—how the pooled result changes in magnitude and heterogeneity—but not to identify the source of heterogeneity.

5.Line 191 (table 2), Put the number of studies pooled for each category. A minimum of 10 studies is required to conduct subgroup analysis, meta-regression, publication bias, and trim-and-fill analysis. https://www.cochrane.org/authors/handbooks-and-manuals/handbook/current/chapter-10

6.Line 191 (table 2), You have conducted sensitivity analysis before striving to capture the source of heterogeneity. Sensitivity analysis is the last step after you have explored the possible sources of heterogeneity through subgroup analysis and meta-regression.

7.line 206(table3), certainty of evidence should be put as individual studies not as a group.

8.Line 217, the 95% CI interval is enough to see the significance, remove the p-value and replace with I-squared value instead. Do the same for the rest of the results.

9.There is variability between sample sizes. However, I haven’t seen meta-regression on sample size to account for the heterogeneity.

**********

what does this mean?). If published, this will include your full peer review and any attached files.

**Do you want your identity to be public for this peer review?**  If you choose “no”, your identity will remain anonymous but your review may still be made public.

For information about this choice, including consent withdrawal, please see our Privacy Policy

Reviewer #1: **Yes:**  Abdulganiyyu A Ibrahim

Reviewer #2: **Yes:**  Eyob Tilahun Abeje

**********

[NOTE: If reviewer comments were submitted as an attachment file, they will be attached to this email and accessible via the submission site. Please log into your account, locate the manuscript record, and check for the action link "View Attachments". If this link does not appear, there are no attachment files.]

Attachment Submitted filename: Review_comments.docx

# Author comment: Author response to Decision Letter 1

**DOI:** 10.1371/journal.pgph.0007191.r002

27 May 2026

Attachment Submitted filename: Response to Reviewers.docx

# Peer review: Decision Letter 1

**DOI:** 10.1371/journal.pgph.0007191.r003

**Authors:** Zulkarnain Jaafar

17 Jun 2026

PGPH-D-26-00622R1

Effects of community health worker–led interventions on cardiometabolic outcomes in vulnerable populations: A systematic review and meta-analysis

PLOS Global Public Health

Dear Dr. Okwah,

Thank you for submitting your manuscript to PLOS Global Public Health. After careful consideration, we feel that it has merit but does not fully meet PLOS Global Public Health’s publication criteria as it currently stands. Therefore, we invite you to submit a revised version of the manuscript that addresses the points raised during the review process.

==============================

EDITOR:Dear Author, this manuscript still requiring major revision. Please make necessary corrections based on the comments provided by the reviewers.

==============================

Please submit your revised manuscript by 15th September 2026. If you will need more time than this to complete your revisions, please reply to this message or contact the journal office at globalpubhealth@plos.org. When you're ready to submit your revision, log on to https://www.editorialmanager.com/pgph/ and select the 'Submissions Needing Revision' folder to locate your manuscript file.

Please include the following items when submitting your revised manuscript:

- A letter that responds to each point raised by the editor and reviewer(s). You should upload this letter as a separate file labeled 'Response to Reviewers'.
- A marked-up copy of your manuscript that highlights changes made to the original version. You should upload this as a separate file labeled 'Revised Manuscript with Track Changes'.
- An unmarked version of your revised paper without tracked changes. You should upload this as a separate file labeled 'Manuscript'.

Guidelines for resubmitting your figure files are available below the reviewer comments at the end of this letter.

As the corresponding author, your ORCID iD is verified in the submission system and will appear in the published article. PLOS supports the use of ORCID, and we encourage all coauthors to register for an ORCID iD and use it as well. Please encourage your coauthors to verify their ORCID iD within the submission system before final acceptance, as unverified ORCID iDs will not appear in the published article. *Only*  the individual author can complete the verification step; PLOS staff *cannot*  verify ORCID iDs on behalf of authors.

We look forward to receiving your revised manuscript.

Kind regards,

Zulkarnain Jaafar

Academic Editor

PLOS Global Public Health

Journal Requirements:

If the reviewer comments include a recommendation to cite specific previously published works, please review and evaluate these publications to determine whether they are relevant and should be cited. There is no requirement to cite these works unless the editor has indicated otherwise.

Additional Editor Comments (if provided):

[Note: HTML markup is below. Please do not edit.]

Reviewers' comments:

Reviewer's Responses to Questions

**Comments to the Author**

Reviewer #1: (No Response)

Reviewer #2: All comments have been addressed

**********

publication criteria?

Reviewer #1: Partly

Reviewer #2: Yes

**********

3. Has the statistical analysis been performed appropriately and rigorously?

Reviewer #1: No

Reviewer #2: Yes

**********

4. Have the authors made all data underlying the findings in their manuscript fully available (please refer to the Data Availability Statement at the start of the manuscript PDF file)?

The PLOS Data policy

Reviewer #1: Yes

Reviewer #2: Yes

**********

5. Is the manuscript presented in an intelligible fashion and written in standard English?

Reviewer #1: Yes

Reviewer #2: Yes

**********

Reviewer #1: Manuscript ID:PGPH-D-25-02592R1

Title:Post-pandemic prevalence of SARS-CoV-2 co-infection in people with Tuberculosis and HIV in Pakistan: Implications for preparedness and response

General Assessment

This manuscript presents findings from a cross-sectional analysis nested within a prospective cohort of newly diagnosed tuberculosis (TB) and HIV patients in Balochistan, Pakistan. The study reports a prevalence of RT-PCR-confirmed SARS-CoV-2 infection of 5.9% among TB patients and 7.4% among people living with HIV (PLHIV), identifies selected correlates of positivity, and discusses implications for integrated pandemic preparedness.

The topic is relevant to global public health and aligns well with the journal's scope. The study addresses an understudied population and provides data from a high TB-burden, resource-constrained setting. The manuscript is generally well organized and substantially improved compared with a typical initial submission. However, several conceptual, methodological, and interpretative issues remain that should be addressed before publication.

The primary concern is that the manuscript occasionally overinterprets cross-sectional SARS-CoV-2 positivity as evidence of a substantial "hidden burden" of COVID-19 and makes comparisons with national surveillance estimates that are not methodologically comparable. Additionally, several statistical and epidemiological issues require clarification.

Major Comments

1. Interpretation of the Primary Finding is Overstated

The central conclusion is that SARS-CoV-2 prevalence was higher among individuals with TB and HIV than in the general population. The abstract explicitly states that prevalence was higher than WHO surveillance estimates reported for Pakistan.

This comparison is problematic because:

* The study population underwent systematic RT-PCR testing irrespective of symptoms.

* National surveillance data arise from routine testing systems with different testing indications.

* The denominators are fundamentally different.

* The study population is restricted to newly diagnosed TB and HIV patients from two hospitals in Balochistan.

Therefore, the manuscript cannot support conclusions that infection risk is necessarily higher than in the general Pakistani population. The authors should consistently describe these findings as occurring in a highly selected clinical population rather than implying population-level excess risk.

Recommendation

Revise the abstract, discussion, and conclusion to avoid direct epidemiological comparisons that imply relative risk.

2. Cross-Sectional Design Does Not Support Causal Interpretation

The manuscript identifies age, cavitary disease, and vaccination as independent correlates of SARS-CoV-2 positivity among TB patients.

However:

* Exposure and outcome were measured simultaneously.

* Temporality cannot be established.

* The study cannot determine whether cavitation predisposes to infection, whether infection influenced radiographic findings, or whether both are linked to unmeasured confounding.

The language in several sections should be revised to emphasize association rather than risk factors.

### Recommendation

Replace "risk factors" with "factors associated with SARS-CoV-2 positivity" throughout the manuscript.

3. Serious Concern Regarding Vaccination Interpretation

Vaccination was associated with greater SARS-CoV-2 positivity among TB patients.

The discussion attributes this finding largely to waning immunity.

While plausible, alternative explanations are equally possible:

* Confounding by healthcare access.

* Occupational exposure.

* Survivor bias.

* Differential health-seeking behavior.

* Chance findings due to small sample size.

Because only 33 TB patients were SARS-CoV-2 positive, the adjusted estimate is unstable.

Recommendation

The discussion should explicitly state that the observed association should not be interpreted as evidence that vaccination increased susceptibility to infection.

4. Limited Statistical Power

Only 42 participants tested positive overall (33 TB and 9 HIV).

This small number of events substantially limits:

* Logistic regression stability.

* Precision of odds ratios.

* Ability to detect associations.

* Interpretation of subgroup analyses.

For example, HIV analyses are based on only 9 positive cases.

The manuscript should discuss possible overfitting of regression models and provide the number of variables included in the final multivariable model.

5. Potential Selection Bias

Participants were recruited only from two facilities in Quetta.

This raises concerns regarding:

* Representativeness of TB patients in Pakistan.

* Representativeness of PLHIV nationally.

* Referral bias.

* Healthcare access bias.

The discussion should more explicitly address the limited external validity of findings.

6. Missing Information on Serology Subsample

Serological results were available for only 341 participants (50% of the cohort).

The manuscript does not explain:

* Why only half were tested.

* Whether tested participants differed systematically from non-tested participants.

* Whether serology sampling was random.

Without this information, interpretation of seroprevalence estimates is difficult.

Minor Comments

1. Terminology

The manuscript alternates between:

* COVID-19 infection

* SARS-CoV-2 infection

* COVID-19 co-infection

These terms are not interchangeable.

Because the outcome was RT-PCR positivity, the preferred terminology should be:

> "SARS-CoV-2 infection" or "SARS-CoV-2 positivity"

throughout the manuscript.

2. Clarify Sample Size Discrepancy

The manuscript reports:

* 562 TB patients and 121 PLHIV enrolled.

* The abstract reports 558 TB patients and 122 PLHIV.

These discrepancies should be reconciled.

3. Chest X-Ray Analysis

Only 369 participants had chest radiographs available.

The manuscript should explain:

* Why imaging was unavailable for many participants.

* Whether missingness could bias the observed cavitation association.

4. HIV Cohort Interpretation

The manuscript states that younger PLHIV may be equally vulnerable to COVID-19 infection.

Given that only nine positive cases occurred, this statement is not adequately supported.

A more cautious interpretation is warranted.

5. Data Availability Statement

The manuscript states that all data are included in the article or supporting information.

The editors should verify that the underlying dataset and supporting tables required for reproducibility are actually available and comply with PLOS data-sharing requirements.

6. Language and Editorial Corrections

Examples include:

* "with aim to supports" should be "with the aim of supporting."

* "need to be prioritized" instead of "need to prioritized."

* "every year kill more people" should be "kills more people."

A thorough copy-edit is recommended.

Strengths

1. Important public health topic relevant to TB/HIV-endemic settings.

2. Prospective cohort infrastructure with standardized enrollment procedures.

3. Systematic RT-PCR testing of all enrolled participants.

4. Inclusion of clinical, laboratory, radiographic, and serologic data.

5. Valuable evidence from a vulnerable and underserved population in Balochistan.

Editorial Recommendation

Reviewer #2: Th authors have addressed the previous comments well. Here are additional comments that needs to be addressed.

1.Line 98 states that high-income countries were also included in this study; however, the inclusion criteria in Line 126 specify that eligible studies specifically involved vulnerable or underserved populations. This is also reflected in the results section (Lines 191–205). Please be transparent about whether high-income countries were included in all search and inclusion processes unless stick to LMIC.

2.Use a consistent referencing style throughout the document. In most sections, square brackets [] are used, whereas in Line 149, parentheses () are used. Please ensure a uniform citation format is maintained consistently across the entire document.

3.Lines 149–151: Does the revised RoB-2 assess blinding and loss to follow-up? Please refer to the Cochrane Library, as these aspects have been updated in the revised version.

4.Table 2: The value (~99%) should be presented in two digits and the tilde (~) should be removed.

5.Lines 459–460: Where is the pooled effect size supporting the statement that “Community health workers are representatives of cultural diversity and represent a scalable and culturally effective approach”? Although this may be true, it is not supported by the pooled findings in this analysis. Please ensure the discussion and conclusion remains strictly aligned with the reported results.

**********

what does this mean?). If published, this will include your full peer review and any attached files.

**Do you want your identity to be public for this peer review?**  If you choose “no”, your identity will remain anonymous but your review may still be made public.

For information about this choice, including consent withdrawal, please see our Privacy Policy

Reviewer #1: **Yes:**  Dr. Abdulganiyyu A Ibrahim

Reviewer #2: **Yes:**  Eyob Tilahun Abeje

**********

[NOTE: If reviewer comments were submitted as an attachment file, they will be attached to this email and accessible via the submission site. Please log into your account, locate the manuscript record, and check for the action link "View Attachments". If this link does not appear, there are no attachment files.]

Attachment Submitted filename: Review round 2.docx

# Author comment: Author response to Decision Letter 2

**DOI:** 10.1371/journal.pgph.0007191.r004

17 Jun 2026

Attachment Submitted filename: Response_to_Reviewers_auresp_2.docx

# Peer review: Decision Letter 2

**DOI:** 10.1371/journal.pgph.0007191.r005

**Authors:** Zulkarnain Jaafar

7 Jul 2026

PGPH-D-26-00622R2

Effects of community health worker–led interventions on cardiometabolic outcomes in vulnerable populations: A systematic review and meta-analysis

PLOS Global Public Health

Dear Dr. Okwah,

Thank you for submitting your manuscript to PLOS Global Public Health. After careful consideration, we feel that it has merit but does not fully meet PLOS Global Public Health’s publication criteria as it currently stands. Therefore, we invite you to submit a revised version of the manuscript that addresses the points raised during the review process.

==============================

EDITOR: Dear Author, please make necessary corrections based on the comments provided by the reviewers.

==============================

Please submit your revised manuscript by 5th October 2026. If you will need more time than this to complete your revisions, please reply to this message or contact the journal office at globalpubhealth@plos.org. When you're ready to submit your revision, log on to https://www.editorialmanager.com/pgph/ and select the 'Submissions Needing Revision' folder to locate your manuscript file.

Please include the following items when submitting your revised manuscript:

- A letter that responds to each point raised by the editor and reviewer(s). You should upload this letter as a separate file labeled 'Response to Reviewers'.
- A marked-up copy of your manuscript that highlights changes made to the original version. You should upload this as a separate file labeled 'Revised Manuscript with Track Changes'.
- An unmarked version of your revised paper without tracked changes. You should upload this as a separate file labeled 'Manuscript'.

Guidelines for resubmitting your figure files are available below the reviewer comments at the end of this letter.

As the corresponding author, your ORCID iD is verified in the submission system and will appear in the published article. PLOS supports the use of ORCID, and we encourage all coauthors to register for an ORCID iD and use it as well. Please encourage your coauthors to verify their ORCID iD within the submission system before final acceptance, as unverified ORCID iDs will not appear in the published article. *Only*  the individual author can complete the verification step; PLOS staff *cannot*  verify ORCID iDs on behalf of authors.

We look forward to receiving your revised manuscript.

Kind regards,

Zulkarnain Jaafar

Academic Editor

PLOS Global Public Health

Journal Requirements:

1. If the reviewer comments include a recommendation to cite specific previously published works, please review and evaluate these publications to determine whether they are relevant and should be cited. There is no requirement to cite these works unless the editor has indicated otherwise.

Additional Editor Comments (if provided):

[Note: HTML markup is below. Please do not edit.]

Reviewers' comments:

Reviewer's Responses to Questions

**Comments to the Author**

Reviewer #1: (No Response)

Reviewer #2: All comments have been addressed

**********

publication criteria?

Reviewer #1: (No Response)

Reviewer #2: Yes

**********

3. Has the statistical analysis been performed appropriately and rigorously?

Reviewer #1: N/A

Reviewer #2: Yes

**********

4. Have the authors made all data underlying the findings in their manuscript fully available (please refer to the Data Availability Statement at the start of the manuscript PDF file)?

The PLOS Data policy

Reviewer #1: Yes

Reviewer #2: Yes

**********

5. Is the manuscript presented in an intelligible fashion and written in standard English?

Reviewer #1: Yes

Reviewer #2: Yes

**********

Reviewer #1: Peer Review Report

Manuscript Title: Community Health Worker–Led Interventions for Cardiometabolic Disease Outcomes in Vulnerable Populations: A Systematic Review and Meta-analysis

Overall Assessment

This manuscript presents a systematic review and meta-analysis of 30 randomized controlled trials involving 15,295 participants assessing the effectiveness of community health worker (CHW)-led interventions on cardiometabolic outcomes among vulnerable populations.

The topic is highly relevant to global public health, particularly given the increasing burden of cardiometabolic diseases (CMDs) in low- and middle-income countries (LMICs) and underserved populations. The manuscript addresses an important evidence gap by synthesizing data across multiple cardiometabolic conditions rather than focusing exclusively on hypertension or diabetes.

The review demonstrates several strengths, including adherence to PRISMA guidance, inclusion of randomized trials only, use of RoB 2 and GRADE methodologies, and exploration of geographic and income-based subgroup effects.

However, significant methodological and interpretive concerns limit confidence in the conclusions. Most notably, the extremely high heterogeneity (I² ≈ 99% across nearly all outcomes), the broad operationalization of vulnerability, potential issues with study classification, and the overinterpretation of subgroup findings require substantial revision.

Major Strengths

1. High Public Health Relevance

The burden of CMDs among vulnerable populations is a major global health concern. The manuscript appropriately situates CHW interventions as potentially scalable approaches to improving prevention and management.

2. Rigorous Study Design

The authors restricted inclusion to randomized controlled trials and followed established systematic review methods.

Strengths include:

• Multiple database search

• Independent screening

• Independent data extraction

• RoB 2 assessment

• GRADE evaluation

3. Inclusion of Diverse Settings

The inclusion of studies from:

• Africa

• Asia

• Latin America

• North America

improves the global relevance of the findings.

4. Policy-Oriented Perspective

The discussion appropriately highlights implications for task-sharing, primary care integration, and WHO HEARTS implementation.

Major Concerns

1. Extreme Heterogeneity Undermines Pooled Estimates

This is the most important concern.

For virtually every outcome:

• SBP: I² = 99.1%

• DBP: I² = 98.6%

• BMI: I² = 98.6%

• LDL-C: I² = 98.7%

• HbA1c: I² = 98.8%

Such levels of heterogeneity are extraordinarily high, raising questions about whether pooled summary estimates are meaningful.

The manuscript repeatedly argues that heterogeneity reflects contextual variability rather than inconsistency. However, this interpretation is insufficiently justified.

Required Revision

The authors should:

• Present prediction intervals for all primary outcomes.

• Conduct more extensive moderator analyses.

• Explore intervention intensity, duration, CHW training, and disease type as sources of heterogeneity.

• Discuss whether pooling was appropriate at all

2. Inappropriate GRADE Assessment of Inconsistency

The manuscript states:

"Inconsistency was not downgraded" despite substantial heterogeneity.

This decision is difficult to justify.

GRADE guidance generally considers unexplained heterogeneity a reason for downgrading certainty.

Given I² values approaching 99%, the rationale for retaining "moderate certainty" evidence appears weak.

Required Revision

Provide a detailed GRADE justification and reconsider certainty ratings.

3. Broad and Potentially Problematic Definition of Vulnerability

The review defines vulnerable populations using multiple criteria, including:

• LMIC residence

• Minority status

• Low SES

• Rural residence

• Marginalized populations

This definition is extremely broad.

Consequently, the review combines:

• U.S. Latino populations

• Filipino Americans

• Rural hypertensive populations in Nepal

• Low-income Argentinians

• African rural populations

into a single pooled estimate.

These groups differ substantially in social context, healthcare access, and disease burden.

Required Revision

Provide a stronger theoretical justification for combining these populations.

4. Subgroup Conclusions Exceed Statistical Evidence

The discussion emphasizes greater effectiveness in LMICs and Africa.

However:

• Region meta-regression was non-significant (p=0.144)

• Income meta-regression was non-significant (p=0.18)

The manuscript nevertheless repeatedly interprets these subgroup differences as meaningful.

This risks ecological overinterpretation.

Required Revision

Temper conclusions and clearly distinguish exploratory findings from statistically supported subgroup effects.

5. Publication Bias Interpretation Is Inconsistent

The authors report:

• Non-significant Egger tests

• Trim-and-fill attenuation to non-significance for several outcomes

Yet the discussion largely dismisses trim-and-fill findings.

Given the extreme heterogeneity, both methods are imperfect.

Required Revision

Present a more balanced interpretation of publication bias uncertainty.

6. Clinical Significance Requires Greater Discussion

The pooled effects are statistically significant but modest:

• SBP: −3.72 mmHg

• DBP: −2.22 mmHg

• BMI: −0.39 kg/m²

• HbA1c: −0.31%

While potentially important at population level, the clinical significance for individual patients deserves more nuanced discussion.

7. Search Strategy Transparency

The search strategy description is overly brief.

Only a simplified example is provided:

"community health worker" AND "cardiometabolic diseases" AND "community-based interventions"

Required Revision

Include the complete search strings in supplementary materials and ensure reproducibility.

Minor Concerns

1. Terminology

The manuscript alternates between:

• community-based interventions

• community health worker interventions

• CHW-led interventions

These should be standardized.

2. References

Several citations supporting claims about global CMD burden appear to rely on future-dated reports (2025–2026).

The authors should ensure that all cited sources were publicly available at the time of manuscript preparation and verify publication details.

3. Discussion Overstates Generalizability

The manuscript states:

"The inclusion of 30 RCTs across multiple regions strengthens the generalizability of results."

Given:

• extremely high heterogeneity,

• diverse intervention models,

• varied populations,

This statement should be qualified.

4. Intervention Characterization

The review would benefit from a table detailing:

• CHW training duration

• supervision structure

• intervention frequency

• intervention intensity

These variables are likely major sources of heterogeneity.

Specific Questions for the Authors

1. Why was inconsistency not downgraded in GRADE despite I² values approaching 99%?

2. Were prediction intervals calculated? If not, why?

3. How were cluster-randomized trials adjusted statistically?

4. Were intervention characteristics evaluated as moderators?

5. How robust are conclusions after trim-and-fill attenuation?

6. What proportion of included studies were judged high risk of bias versus "some concerns"?

Reviewer #2: The authors have addressed the previous comments; however, some conclusions remain overstated and are not supported by the findings. This issue needs to be addressed.

1.Abstract: Try to reduce abbreviations in the abstract. For variables written in abbreviation, provide the full form at least once (e.g., LMIC, HICs).

2. Data availability statement: You have generated new datasets from primary studies. Therefore, check whether your results are correctly reflected in the data availability statement.

3. S2 File 2 (Search strategy): You have included only the search strings, but you need to be transparent and provide all necessary information about the search strategy in a tabular format, including:

● The database name

● The search strings used in that database

● The date when the search was conducted

● The number of studies identified

4. “Equivalent search strategies were adapted for Scopus, Embase, Cochrane CENTRAL, and ClinicalTrials.gov.” In Supplementary File 2, how were they equivalent? Clearly present the search strategy for each database.

5. Line 113: Remove vague words such as “inception.”

6. In the Data Extraction and Quality Assessment section, you have abbreviated the roles of extractors and discrepancy handlers, but not in the Literature Search and Eligibility Criteria sections. Please maintain consistency.

7. Lines 439–452: Where in your manuscript have you assessed the link between follow-up duration and improvement in your outcome variables? Your recommendations (future directions) should be aligned with your findings.

8. Lines 461–463: Instead of using general terms like “contextual and health system factors,” be clear and specific in your conclusion. State exactly what you have found.

**********

what does this mean?). If published, this will include your full peer review and any attached files.

**Do you want your identity to be public for this peer review?**  If you choose “no”, your identity will remain anonymous but your review may still be made public.

For information about this choice, including consent withdrawal, please see our Privacy Policy

Reviewer #1: **Yes:**  Dr. Abdulganiyyu A Ibrahim

Reviewer #2: **Yes:**  Eyob Tilahun Abeje

**********

[NOTE: If reviewer comments were submitted as an attachment file, they will be attached to this email and accessible via the submission site. Please log into your account, locate the manuscript record, and check for the action link "View Attachments". If this link does not appear, there are no attachment files.]

Attachment Submitted filename: PGPH revison 2 review.docx

# Author comment: Author response to Decision Letter 3

**DOI:** 10.1371/journal.pgph.0007191.r006

15 Jul 2026

Attachment Submitted filename: Response_to_Reviewers.docx

# Peer review: Decision Letter 3

**DOI:** 10.1371/journal.pgph.0007191.r007

**Authors:** Zulkarnain Jaafar

4 Aug 2026

PGPH-D-26-00622R3

Effects of community health worker–led interventions on cardiometabolic outcomes in vulnerable populations: A systematic review and meta-analysis

PLOS Global Public Health

Dear Dr. Okwah,

Thank you for submitting your manuscript to PLOS Global Public Health. After careful consideration, we feel that it has merit but does not fully meet PLOS Global Public Health’s publication criteria as it currently stands. Therefore, we invite you to submit a revised version of the manuscript that addresses the points raised during the review process.

==============================

EDITOR: Dear Author, please make necessary revisions based on the comments provided by the reviewer.

==============================

Please submit your revised manuscript by 2nd September 2026. If you will need more time than this to complete your revisions, please reply to this message or contact the journal office at globalpubhealth@plos.org. When you're ready to submit your revision, log on to https://www.editorialmanager.com/pgph/ and select the 'Submissions Needing Revision' folder to locate your manuscript file.

Please include the following items when submitting your revised manuscript:

- A letter that responds to each point raised by the editor and reviewer(s). You should upload this letter as a separate file labeled 'Response to Reviewers'.
- A marked-up copy of your manuscript that highlights changes made to the original version. You should upload this as a separate file labeled 'Revised Manuscript with Track Changes'.
- An unmarked version of your revised paper without tracked changes. You should upload this as a separate file labeled 'Manuscript'.

Guidelines for resubmitting your figure files are available below the reviewer comments at the end of this letter.

As the corresponding author, your ORCID iD is verified in the submission system and will appear in the published article. PLOS supports the use of ORCID, and we encourage all coauthors to register for an ORCID iD and use it as well. Please encourage your coauthors to verify their ORCID iD within the submission system before final acceptance, as unverified ORCID iDs will not appear in the published article. *Only*  the individual author can complete the verification step; PLOS staff *cannot*  verify ORCID iDs on behalf of authors.

We look forward to receiving your revised manuscript.

Kind regards,

Zulkarnain Jaafar

Academic Editor

PLOS Global Public Health

Journal Requirements:

1. If the reviewer comments include a recommendation to cite specific previously published works, please review and evaluate these publications to determine whether they are relevant and should be cited. There is no requirement to cite these works unless the editor has indicated otherwise.

2. Please review your reference list to ensure that it is complete and correct. If you have cited papers that have been retracted, please include the rationale for doing so in the manuscript text, or remove these references and replace them with relevant current references. Any changes to the reference list should be mentioned in the rebuttal letter that accompanies your revised manuscript. If you need to cite a retracted article, indicate the article’s retracted status in the References list and also include a citation and full reference for the retraction notice.

Additional Editor Comments (if provided):

[Note: HTML markup is below. Please do not edit.]

Reviewers' comments:

Reviewer's Responses to Questions

**Comments to the Author**

Reviewer #1: All comments have been addressed

Reviewer #2: All comments have been addressed

**********

publication criteria?

Reviewer #1: Yes

Reviewer #2: Yes

**********

3. Has the statistical analysis been performed appropriately and rigorously?

Reviewer #1: Yes

Reviewer #2: Yes

**********

4. Have the authors made all data underlying the findings in their manuscript fully available (please refer to the Data Availability Statement at the start of the manuscript PDF file)?

The PLOS Data policy

Reviewer #1: Yes

Reviewer #2: Yes

**********

5. Is the manuscript presented in an intelligible fashion and written in standard English?

Reviewer #1: Yes

Reviewer #2: Yes

**********

Reviewer #1: Manuscript Title

Effects of Community Health Worker–Led Interventions on Cardiometabolic Outcomes in Vulnerable Populations: A Systematic Review and Meta-analysis

Overall Assessment

The authors have responded constructively to the major concerns raised during the previous review cycle. The revised manuscript demonstrates substantial improvement in methodological transparency, interpretation of heterogeneity, GRADE assessment, reporting of uncertainty, and reproducibility.

Importantly, the authors have:

• Added prediction intervals for all outcomes.

• Revised GRADE certainty ratings to account for unexplained heterogeneity.

• Expanded discussion of limitations and generalizability.

• Added intervention-characteristic reporting.

• Tempered subgroup interpretations and reclassified them as hypothesis-generating.

Overall, the manuscript is considerably stronger than the previous version and now provides a more balanced interpretation of findings. Nevertheless, several issues remain that should be addressed before acceptance.

Major Improvements Since Previous Version

1. Prediction Intervals Appropriately Added

One of the principal weaknesses of the prior version was the absence of prediction intervals despite extreme heterogeneity.

The revised manuscript now reports prediction intervals for all major outcomes, including:

• SBP: −15.12 to 7.67 mmHg

• HbA1c: −1.02% to 0.50%

These intervals appropriately demonstrate uncertainty regarding expected effects in future settings.

This revision substantially improves interpretability.

2. GRADE Assessment Is More Appropriate

The prior version did not adequately downgrade for inconsistency despite I² values approaching 99%.

The authors have corrected this issue and now downgrade:

• SBP

• DBP

• HbA1c

for inconsistency when heterogeneity remained unexplained.

This represents a significant methodological improvement.

3. Better Discussion of Generalizability

The revised manuscript now explicitly acknowledges that:

substantial heterogeneity limits generalizability to any single setting and prediction intervals cross the null for all outcomes.

This is a much more balanced interpretation than in the previous version.

4. Improved Reproducibility

The addition of:

• complete search strategies,

• supplementary intervention-characteristic tables,

meaningfully strengthens transparency.

Remaining Concerns

1. Persistent Overinterpretation of LMIC and Regional Subgroup Findings

Although the authors have moderated their language, residual overinterpretation remains.

The Discussion still states:

“Several mechanisms may explain the stronger blood pressure reductions observed in LMICs and certain geographic regions.”

However, earlier responses acknowledge that:

• region meta-regression was not significant,

• income meta-regression was not significant.

The current text risks implying causal explanations for subgroup differences that were not statistically confirmed.

Recommendation

Revise wording to emphasize that these explanations remain speculative and exploratory.

2. Contradictory Interpretation of Heterogeneity

The manuscript still contains language suggesting that heterogeneity should not necessarily be viewed as a limitation:

“heterogeneity should not be viewed as a limitation that undermines effectiveness”

This statement is problematic.

While contextual heterogeneity is expected in complex interventions, I² values of 96–99% remain a serious limitation to confidence in pooled effects.

Recommendation

Reframe this paragraph more cautiously to avoid minimizing the implications of extreme heterogeneity.

3. Abstract Certainty Ratings Appear Inconsistent

The tracked-changes version indicates:

“lowmoderate” for SBP and DBP and “moderate” for BMI.

Meanwhile Table 3 reports:

• SBP = Low

• DBP = Low

• BMI = Moderate

• HbA1c = Very low

• LDL-C = Low

Recommendation

Carefully proofread the abstract and ensure consistency between abstract, results, and Table 3.

4. Some Residual Editorial Artifacts Remain

Several sections appear to retain tracked-change artifacts or duplicated text, for example:

• “CHW-ledCommunity-based interventions”

• “improved systolic blood pressure ... However” formatting inconsistencies

• duplicated certainty language in Section 3.3

These likely arose during revision and should be corrected before production.

5. Reference Verification

Most citation concerns have been addressed.

However, several references remain future-dated (2025–2026).

If accepted, the editorial office should ensure:

• all references are formally published,

• citation details are accurate,

• prepublication references are properly identified.

Minor Comments

Methods

The manuscript now reports that:

• 12/30 studies were low risk,

• 18/30 had some concerns,

• none were high risk.

This addition is valuable and should be retained in the main text.

Intervention Characterization

The new supplementary intervention table is helpful.

Consider briefly summarizing the range of intervention durations and contact frequencies within the Results section.

Conclusion

The revised conclusion appropriately states that findings are:

“hypothesis-generating rather than practice-changing”

This is an important improvement and should remain.

Reviewer #2: (No Response)

**********

what does this mean?). If published, this will include your full peer review and any attached files.

**Do you want your identity to be public for this peer review?**  If you choose “no”, your identity will remain anonymous but your review may still be made public.

For information about this choice, including consent withdrawal, please see our Privacy Policy

Reviewer #1: **Yes:**  Dr. Ibrahim A. Abdulganiyyu

Reviewer #2: **Yes:**  Eyob Tilahun Abeje

**********

[NOTE: If reviewer comments were submitted as an attachment file, they will be attached to this email and accessible via the submission site. Please log into your account, locate the manuscript record, and check for the action link "View Attachments". If this link does not appear, there are no attachment files.]

# Author comment: Author response to Decision Letter 4

**DOI:** 10.1371/journal.pgph.0007191.r008

4 Aug 2026

Attachment Submitted filename: Response_to_Reviewers_auresp_4.docx

# Editor report: Decision Letter 4

**DOI:** 10.1371/journal.pgph.0007191.r009

**Authors:** Zulkarnain Jaafar

10 Aug 2026

Effects of community health worker–led interventions on cardiometabolic outcomes in vulnerable populations: A systematic review and meta-analysis

PGPH-D-26-00622R4

Dear Okwah,

We are pleased to inform you that your manuscript 'Effects of community health worker–led interventions on cardiometabolic outcomes in vulnerable populations: A systematic review and meta-analysis' has been provisionally accepted for publication in PLOS Global Public Health.

Before your manuscript can be formally accepted you will need to complete some formatting changes, which you will receive in a follow up email. A member of our team will be in touch with a set of requests.

Please note that your manuscript will not be scheduled for publication until you have made the required changes, so a swift response is appreciated.

IMPORTANT: The editorial review process is now complete. PLOS will only permit corrections to spelling, formatting or significant scientific errors from this point onwards. Requests for major changes, or any which affect the scientific understanding of your work, will cause delays to the publication date of your manuscript.

If your institution or institutions have a press office, please notify them about your upcoming paper to help maximize its impact. If they'll be preparing press materials, please inform our press team as soon as possible -- no later than 48 hours after receiving the formal acceptance. Your manuscript will remain under strict press embargo until 2 pm Eastern Time on the date of publication. For more information, please contact globalpubhealth@plos.org.

Thank you again for supporting Open Access publishing; we are looking forward to publishing your work in PLOS Global Public Health.

Best regards,

Zulkarnain Jaafar

Academic Editor

PLOS Global Public Health

***********************************************************

Reviewer Comments (if any, and for reference):
